# Cr(VI) remediation from aqueous environment through modified-TiO_2_-mediated photocatalytic reduction

**DOI:** 10.3762/bjnano.9.137

**Published:** 2018-05-16

**Authors:** Rashmi Acharya, Brundabana Naik, Kulamani Parida

**Affiliations:** 1 Centre for Nano Science and Nano Technology, Siksha ‘O’ Anusandhan University, Bhubaneswar 751030, India

**Keywords:** charge transfer, Cr(VI) reduction, heterojunction, modified TiO_2_, photocatalysis, spinel oxides

## Abstract

Cr(VI) exhibits cytotoxic, mutagenic and carcinogenic properties; hence, effluents containing Cr(VI) from various industrial processes pose threat to aquatic life and downstream users. Various treatment techniques, such as chemical reduction, ion exchange, bacterial degradation, adsorption and photocatalysis, have been exploited for remediation of Cr(VI) from wastewater. Among these, photocatalysis has recently gained considerable attention. The applications of photocatalysis, such as water splitting, CO_2_ reduction, pollutant degradation, organic transformation reactions, N_2_ fixation, etc., towards solving the energy crisis and environmental issues are briefly discussed in the Introduction of this review. The advantages of TiO_2_ as a photocatalyst and the importance of its modification for photocatalytic reduction of Cr(VI) has also been addressed. In this review, the photocatalytic activity of TiO_2_ after modification with carbon-based advanced materials, metal oxides, metal sulfides and noble metals towards reduction of Cr(VI) was evaluated and compared with that of bare TiO_2_. The photoactivity of dye-sensitized TiO_2_ for reduction of Cr(VI) was also discussed. The mechanism for enhanced photocatalytic activity was highlighted and attributed to the resultant properties, namely, effective separation of photoinduced charge carriers, extension of the light absorption range and intensity, increase of the surface active sites, and higher photostability. Advantages and limitations for photoreduction of Cr(VI) over modified TiO_2_ are depicted in the Conclusion. The various challenges that restrict the technology from practical applications in remediation of Cr(VI) from wastewater were addressed in the Conclusion section as well. The future perspectives of the field presented in this review are focused on the development of whole-solar-spectrum responsive, TiO_2_-coupled photocatalysts which provide efficient photocatalytic reduction of Cr(VI) along with their good recoverability and recyclability.

## Review

### Introduction

The increase in the global population demands rapid growth of industrialization and urbanization, which in turn act to increase the level of environmental pollution [1–3]. Heavy metals contribute to a significant extent towards environmental pollution because of their toxicity, bio-accumulation and non-biodegradable nature. They also release large quantities of hazardous waste during their extraction. Hence, removal of toxic heavy metal ions from wastewater is considered as one of the most important environmental issues worldwide.

**Figure 1:**
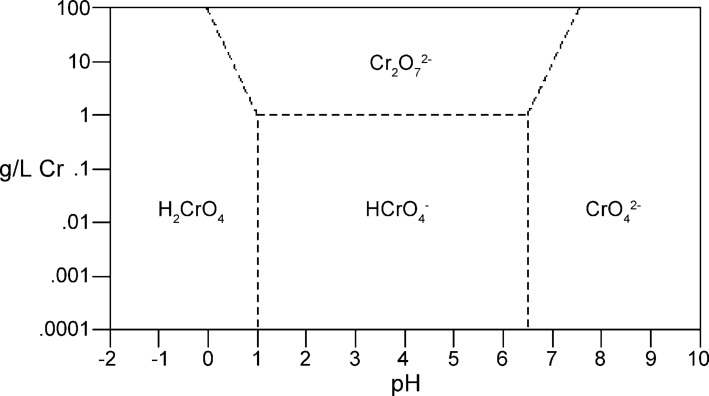
Speciation diagram of Cr(VI). Reprinted from [6], copyright 2016 Thermo Fisher Scientific Inc.

Among the toxic heavy metals, chromium has been a major environmental concern in wastewater treatment. It exists in various oxidation states starting from Cr(II) to Cr(VI). The aqueous environment mostly contains Cr(III) and different Cr(VI) species like HCrO_4_^−^, Cr_2_O_7_^2−^, CrO_4_^2−^ and H_2_CrO_4_. The speciation of these Cr(VI) species depends on pH and concentration of the solution [4–5]. Figure 1 shows the speciation of Cr(VI) at different concentration and pH. It is evident from Figure 1 that HCrO_4_^−^ and Cr_2_O_7_^2−^ are the stable Cr(VI) species between pH 1.0 and 6.0 whereas CrO_4_^2−^ predominates above pH 6.0 [6].

Cr(VI) compounds are corrosion inhibitors and are toxic and are thus mostly used in different industrial processes such as metal plating, leather tanning and pigment manufacturing. Effluents containing a high Cr(VI) concentration from these industries are undesirably discharged into the aquatic environment [7–9]. The chrome tanning process is the most preferred among 80% of tannery industries in India and most of them discharge untreated wastewater into nearby water bodies [4]. The Ganges River at Kanpur city of Uttar Pradesh contains 12.5 mg L^−1^ of Cr(VI) and the concentration of Cr(VI) in surface water in the Ranipet industrial area of Tamilnadu varies from 2.1 to 214 mg L^−1^ because of a number of tanneries are operated in and around these areas [10]. Moreover, accidental leakage and improper disposal at mining sites are also sources of Cr(VI) contamination in natural water ecosystems [11–12].

Being a strong oxidizing agent with cytotoxic, mutagenic and carcinogenic properties, Cr(VI) causes a wide range of clinical/health hazards like damage to liver and kidney, lung carcinoma, nausea, skin dermatitis, nasal membrane inflammation, ulceration, irritation of the gastro-intestinal tract and renal damage, when consumed above the permissible limit. The US Environment Protection Agency (USEPA) has placed it on the priority list of toxic pollutants and has mandated a maximum acceptable concentration of 50 μg L^−1^ in potable water [13–15].

Therefore, it is now of great importance to explore the efficient and economical ways for the treatment of Cr(VI)-rich wastewater. Various techniques, such as chemical reduction, ion exchange, bacterial degradation and adsorption, have been exploited to treat Cr(VI) [16–19]. Among these technologies, chemical reduction has extensively been investigated because it involves conversion of toxic Cr(VI) species to less toxic Cr(III) ions, which are precipitated as green precipitates of Cr(OH)_3_ in neutral or alkaline solutions (Ksp_(Cr(OH)3)_ = 6.3 × 10^−31^] and are removed as solid wastes [20–21]. However, use of this traditional technique is restricted due to high cost and generation of secondary waste as it requires a large amount of reducing agent such as ferrous sulfate, sodium hydrogen sulfite, sodium pyro-sulfite, hydrazine hydrate or sulfur dioxide [22–25].

In contrast, semiconductor-based photocatalysis has received considerable attention worldwide for its diversified potential applications to solve the global energy crisis and environmental issues in a sustainable and ecologically friendly manner [26–29]. This process involves: (i) generation of renewable energy such as H_2_ and O_2_ by photoelectrochemical water splitting [30–32], (ii) photocatalytic CO_2_ conversion [33–37], (iii) photocatalytic nitrogen (N_2_) fixation [38], (iv) selective organic transformation for the ﬁne chemical synthesis [39–42] and (v) photodegradation of pollutants [43–49]. Semiconductor-based photocatalysis proceeds through following three steps: (1) absorption of light; (2) separation and transport of charge carriers; and (3) redox reactions on the surface of the semiconductor.

When photons with energy greater than the band gap energy of the semiconductor photocatalyst (SP) are absorbed, photogenerated electrons are excited to conduction band (CB) leaving behind holes at the valence band (VB) as per Equation 1:

[1]
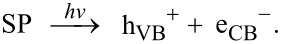


These photogenerated species (electrons (e_CB_^−^) and holes (h_VB_^+^)) must be effectively separated before they can carry out appropriate redox reactions at the semiconductor surface.

### Photoelectrochemical water splitting

Hydrogen (H_2_) is considered as a sustainable, clean and renewable energy source to provide a solution to the global energy crisis [50]. The conventional processes, such as steam reforming, partial oxidation, coal gasification, etc. used for production of H_2_ from fossil fuels (natural gas and coal), are limited because of high cost and stringent environmental regulations [51]. Photocatalytic water splitting for the production of H_2_ is recognized as a green technology since it uses abundantly available water resources and inexhaustible solar energy. Therefore, substantial research in this field has been carried out since the pioneering work of Fujishima and Honda over titanium dioxide (TiO_2_) electrodes under irradiation of ultraviolet (UV) light in 1972 [52]. In photocatalytic water splitting, h_VB_^+^ in the VB oxidize H_2_O to produce O_2_ as shown in Equation 2 only when the band edge potential at the VB is more positive than the oxidation potential of O_2_ evolution (E^0^_O2/H2O_ = 1.23 V vs NHE).

[2]
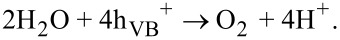


On the other hand, H_2_ gas is produced (Equation 3) at the CB after the reduction reaction carried out by e_CB_^−^ when the CB potential is more negative than the redox potential of H_2_ (E^0^_H+/H2_ = 0 V vs NHE at pH 0.0) .

[3]
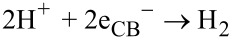


### Photocatalytic CO_2_ conversion

The increasing concentration of greenhouse gases (particularly CO_2_) in the atmosphere has caused environmental issues such as global warming and climate changes. The technologies used to reduce the CO_2_ concentration are energy consuming and expensive [36–37]. In recent years, semiconductor-based visible-light-induced photocatalytic reduction of CO_2_ has emerged as an attractive and viable approach for not only decreasing the concentration of atmospheric CO_2_ but also producing energy fuels such as CH_4_ [53]. In the process of photocatalytic CO_2_ conversion, H_2_O and CO_2_ adsorbed on the surface of the semiconductor are converted to CH_4_ and O_2_ under irradiation of suitable light energy as shown in the following equation.

[4]
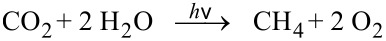


The mechanism of photocatalytic reduction of CO_2_ involves the production of e_CB_^−^ and h_VB_^+^ in the CB and VB, respectively, under irradiation of suitable light energy. CO_2_ is reduced with the help of e_CB_^−^ to CH_4_ at the CB if the minimum CB potential is more negative than the reduction potential of CO_2_/CH_4_ (−0.24 V vs NHE) [54]. Similarly, the oxidation of water takes place by h_VB_^+^ in the VB, only when the maximum VB potential is more positive than the oxidation potential of H_2_O/H^+^ (+0.82 V vs NHE at pH 7.0) [55].

### Photocatalytic nitrogen (N_2_) fixation

The fixation of N_2_ to NH_3_ through semiconductor photocatalysis is gaining attention mostly due to the use of a relatively clean, cheap and easily accessible driving force (light) and ingredients (water and air). During photocatalytic N_2_ fixation, e_CB_^−^ are promoted to the CB, leaving h_VB_^+^ in the VB, upon irradiation with suitable light energy on the semiconductor surface. The h_VB_^+^ so formed in the VB, oxidizes H_2_O to liberate O_2_ with the production of protons (H^+^ ions) if the VB has a more positive potential than that of the potential of O_2_ evolution. With the help of these protons, e_CB_^−^ in the CB, reduces N_2_ molecules adsorbed on the surface of the semiconductor to NH_3_ through a number of step reactions. This occurs only when the CB potential is more negative than the reduction potential of the N_2_/NH_3_ redox couple [38]. The overall photocatalytic N_2_ fixation reaction is shown in Equation 5.

[5]
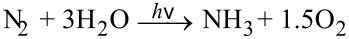


### Selective organic transformation for fine chemical synthesis

Photocatalytic, selective, organic transformations are currently preferred over the conventional processes for synthesis of fine chemicals basically due to two reasons. The first one is to restrict the use of environmentally detrimental chemical reagents such as heavy metal catalysts, oxidizing agents (Cr(VI), MnO_4_^−^, ClO^−^, Cl_2_ etc.) and reducing agents (H_2_, CO). Secondly, energy consuming conditions such as high temperature and high pressure processes are to be avoided [39–42]. In semiconductor-mediated photocatalysis, the e_CB_^−^ in the CB combines with molecular O_2_ as shown in Equation 6 to form a superoxide anion (O_2_^•−^), which acts as a strong oxidizing agent. Similarly, strongly oxidizing hydroxyl radicals (•OH) are produced in the VB by the reaction of h_VB_^+^ with either surface hydroxy groups (–OH) or adsorbed water molecules (Equation 7).

[6]
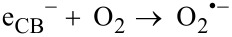


[7]
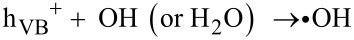


The reaction of these active species with given organic compounds under suitable reaction conditions facilitates the selective organic transformations. For example, Hu et al. demonstrated that photocatalytic, selective oxidation of alcohol to aldehyde can be carried out by h_VB_^+^ and O_2_^•−^ on the surface of CdS/TiO_2_ nanocomposites under visible-light irradiation. The authors have also reported that •CO_2_^−^ radicals (produced by the reaction of h_VB_^+^ with HCO_2_NH_4_) and e_CB_^−^ are responsible for the reduction of 4-nitroaniline to *p*-phenylenediamine over CdS/TiO_2_ photocatalysts [56].

### Photodegradation of pollutants

The principle of photocatalysis for degradation of pollutants was first applied by Frank and Bard in 1977 to reduce CN^−^ in water [57–58]. Thereafter, significant research on photocatalytic degradation of hazardous organic compounds and reduction of toxic heavy metal ions (Cr(VI)) was carried out over various semiconductors upon irradiation of suitable light energy. The mechanism of photodegradation of organic pollutants involves the formation of reactive species like O_2_^•−^ and ^•^OH as per Equation 6 and Equation 7. Some of the O_2_^•−^ species combine with H^+^ ions to form ^•^OOH as represented in Equation 8.

[8]
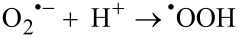


These active species (^•^OH, O_2_^•−^ and ^•^OOH) decompose the organic pollutants to less harmful compounds like H_2_O and CO_2_ (Equation 9).

[9]
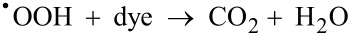


### Photocatalytic reduction of Cr(VI)

**Figure 2:**
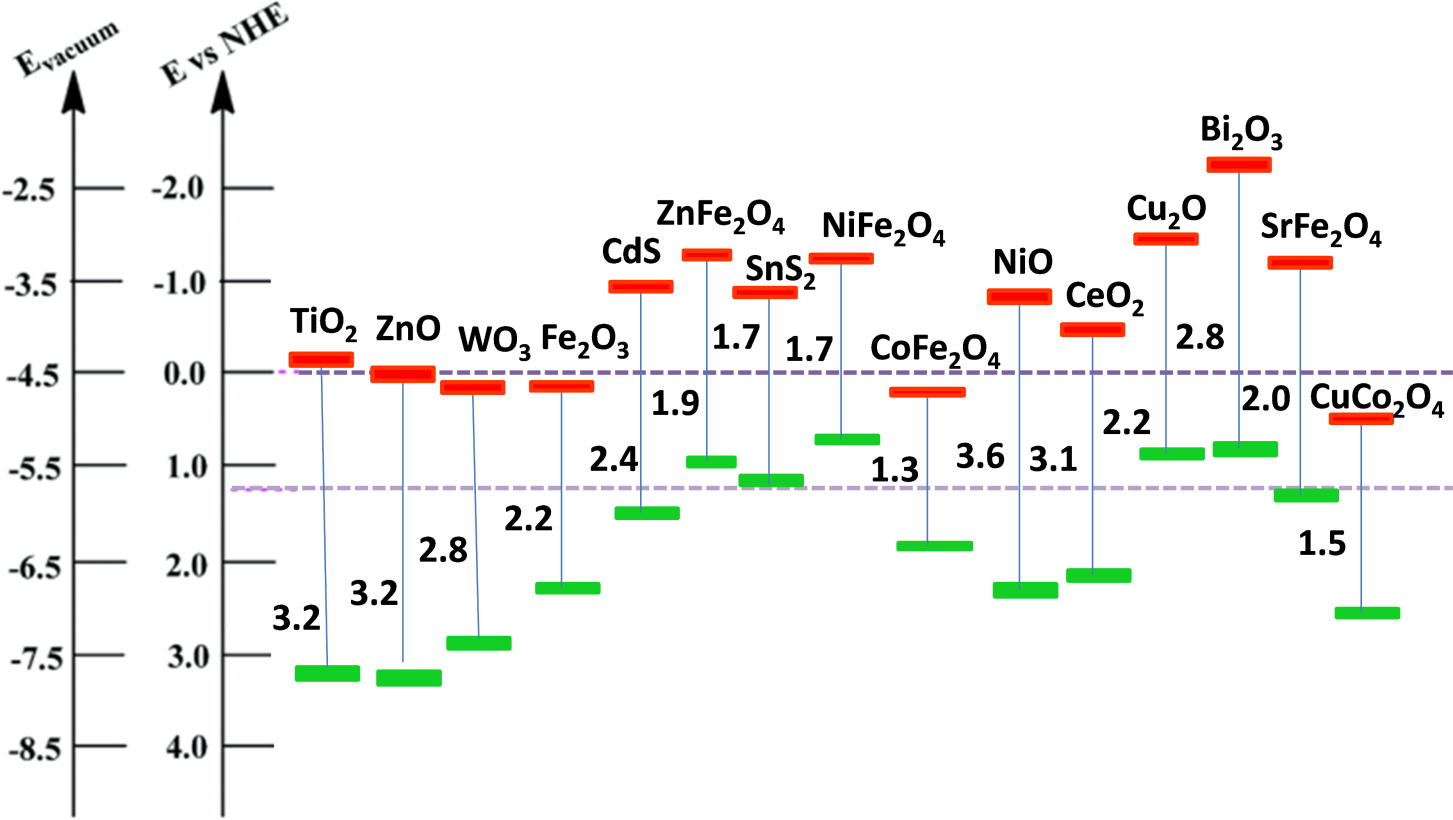
The band edge potentials and band gaps of different semiconductors that combine with TiO_2_ for enhanced photocatalytic reduction of Cr(VI).

The semiconductor-mediated photocatalytic reduction of aqueous Cr(VI) has also recently gained tremendous importance because of its simple operation under ambient conditions, low cost, high efficiency and reusability. It uses renewable and pollution-free solar energy and produces minimal secondary waste without using toxic chemicals that follow the rules of green chemistry [59–61]. Various semiconductor photocatalysts such as CdS, ZnO, WO_3_, SnO_2_, and TiO_2_ have been used for the photocatalytic reduction of aqueous Cr(VI) in recent years. Among them TiO_2_ has extensively been investigated [62–68] due to its nontoxicity, excellent photochemical stability, great oxidizing power, chemical inertness, high abundance, low cost and environmentally friendly nature [69–72]. Moreover, photoexcited TiO_2_ surfaces possess super hydrophilic properties which are evident from their excellent anti-fogging and self-cleaning abilities [73]. The unique feature of TiO_2_ among other semiconductors is that the reduction of Cr(VI) occurs at its CB since the redox potential of Cr(VI) (E^0^_Cr(VI)/Cr(III)_ = 1.33 V in acidic medium) is more positive than the CB potential and the oxidation of water takes place simultaneously at its VB due to the more negative redox potential of H_2_O (E^0^_O2/H2O_) = 1.23 V) than the VB potential (Figure 2) [74].

Therefore, TiO_2_ has widely been accepted by the research community as a green photocatalyst [75]. Irradiation using UV light on TiO_2_ results in the formation of e_CB_^−^ and h_VB_^+^ at its CB and VB, respectively. The e_CB_^−^ reduces Cr(VI) species to Cr(III) as per Equation 10 and h_VB_^+^ oxidizes water to O_2_ (Equation 11).

[10]
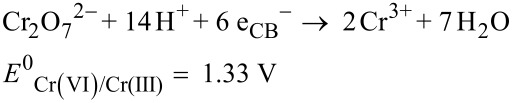


[11]
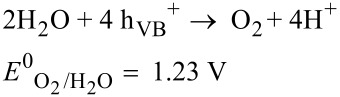


**Figure 3:**
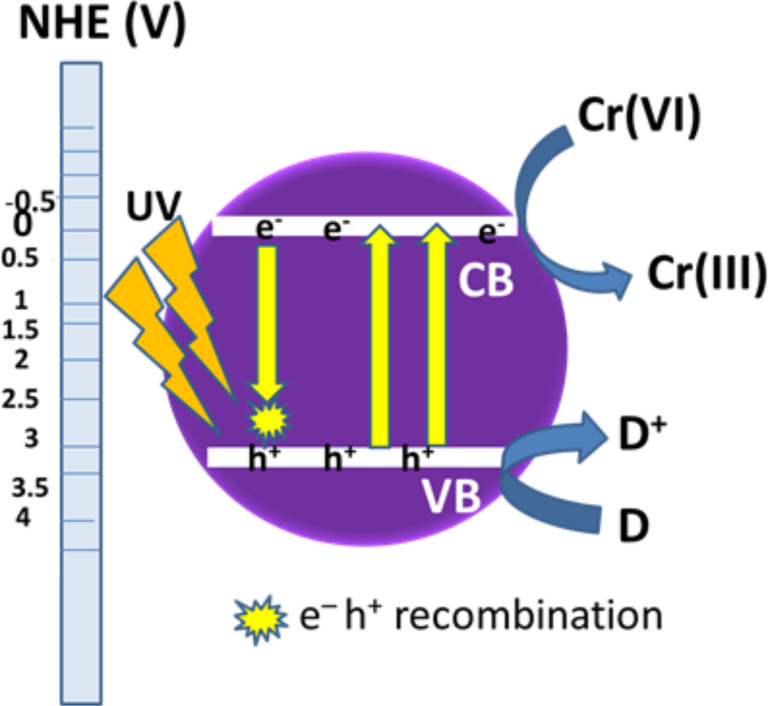
Mechanism of photocatalytic reduction of Cr(VI) over neat TiO_2_. (D = donor, D^+^ = oxidized product).

In some cases, hole scavengers are used to facilitate the electron–hole separation process. However, application of TiO_2_ in photocatalysis is largely restricted mainly due to the following reasons. (i) The wide band gap of 3.2 eV causes excitation of electrons from the valence band under irradiation of UV light, which is only 3% of the total solar radiation, resulting in limited use of pure TiO_2_ in solar energy conversion [76–78]. (ii) The recombination of excited charge carriers in bare TiO_2_ takes place at such a high rate that more than 90% of the recombination processes occur in 10 ns [79], leaving behind a small fraction of the excited carriers to be transferred to the surface of TiO_2_. This low electron transfer rate on the interface and fast recombination of photoinduced charge carriers causes its poor photocatalytic and photoelectrochemical efficiency [80–83]. (iii) The tendency of nanostructured TiO_2_ to agglomerate results in difficulties during the separation process [84]. The detailed mechanism for photocatalytic reduction of Cr(VI) by neat TiO_2_ is presented in Figure 3.

To overcome these limitations, researchers have adopted several modifications such as (i) doping with metals, nonmetals and co-doping [85–88], (ii) coupling of photosensitized nanomaterials [89], (iii) combination of heterojunction materials [90] and (iv) introduction of plasmonic photocatalysts for hot electron generation [62,76]. The modification of TiO_2_ induces the enhancement of photocatalytic activity by achieving a more efficient charge separation, increasing the lifetime of the charge carriers, inhibiting the recombination of electron–hole pairs and facilitating interfacial charge transfer to adsorbed substrates [91–92]. In this review we have focused on modification of nanostructured TiO_2_ with carbon-based advanced materials, noble metals, oxides and sulfides of transition metals for enhanced photocatalytic activity towards degradation of Cr(VI). The photocatalytic reduction of Cr(VI) over dye-sensitized TiO_2_ is also briefly discussed. The present review article has been divided into six sections. The optical and electrochemical characteristics of modified TiO_2_ photocatalysts are discussed in the first section. In the second section, we have reviewed how carbon-based advanced materials like reduced graphene oxide (RGO), carbon nanotubes (CNTs) and carbon dots (CDs) improve the photocatalytic activity and light absorption range of TiO_2_ towards reduction of Cr(VI). The importance of the combination of metal oxides with TiO_2_ for photocatalytic reduction of Cr(VI) was discussed in section three. Section four highlights the enhancement of photocatalytic activity and the light absorption range of TiO_2_ by modification with metal sulfides. The enhancement in photocatalytic reduction of Cr(VI) over noble-metal-modified TiO_2_ is depicted in section five whereas section six includes the use of dye-sensitized TiO_2_ for photoreduction of Cr(VI).

#### Optical and electrochemical characteristics of modified TiO_2_ photocatalysts

The photocatalytic activity of a photocatalyst is characterized by its optical and electrochemical properties. Modifications of titania can hinder the recombination of charge carriers and extend the light absorption range, which are evident from optical and photoelectrochemical studies. Optical studies such as ultraviolet–visible diffuse reflectance spectroscopy (UV–vis DRS) and photoluminescence spectroscopy (PL) explain the shift of the absorption range to the visible region and prohibition of recombination of charge carriers, respectively. The doping of nonmetals such as N, S, or B narrows the band gap either by creating a mid-band gap or shifting the valence band to upper positions, resulting in a redshift. Even modification of titania with semiconductor oxides or sulfides improves the light absorption. It was evident from UV–vis DRS spectra that light absorption is shifted to longer wavelengths when TiO_2_ is combined with SnS_2_ [93]. Moreover, it is seen that modification with sulfates induces a redox couple which facilitates the electron transfer, and hence, better photocatalytic activity. Naik et al. have shown S and N modified titania where electron shuffle takes place by the sulfate redox couple attached to nitrogen-doped TiO_2_ [63]. Hydrogenated defect-promoted black titania exhibits much higher absorption and photocatalytic activity [94–95].

**Figure 4:**
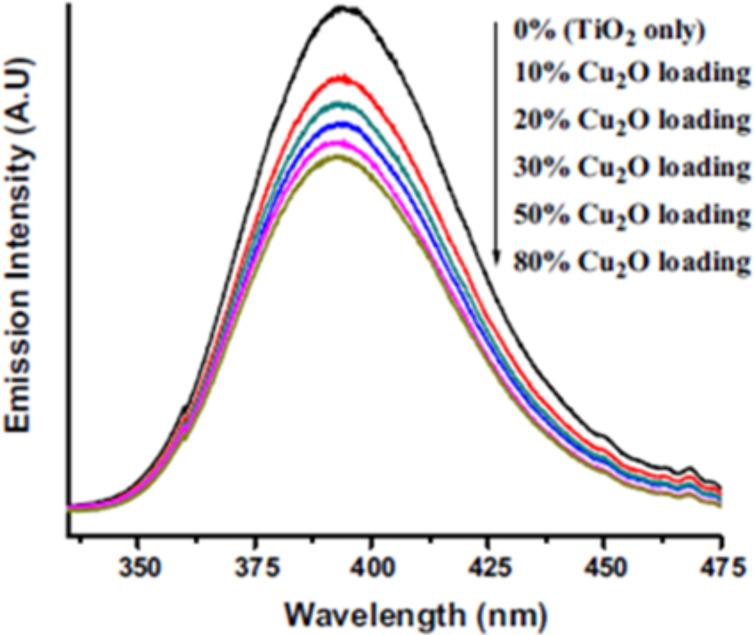
Photoluminescence spectra of bare TiO_2_ and Cu_2_O–TiO_2_ samples. Reprinted from [96], copyright 2016 Springer Science+Business Media.

The recombination and charge transfer efficiency can be understood from PL spectra. The PL emission intensity is related to the recombination of excited electrons and holes. The reduction of the PL emission peak indicates less recombination and higher charge transfer. Modified titania has a greater ability to capture the photogenerated charge carriers for enhanced photocatalytic activity. Figure 4 suggests a higher charge transfer (lower PL peak) of Cu_2_O-modified TiO_2_ than the pure TiO_2_ [96].

**Figure 5:**
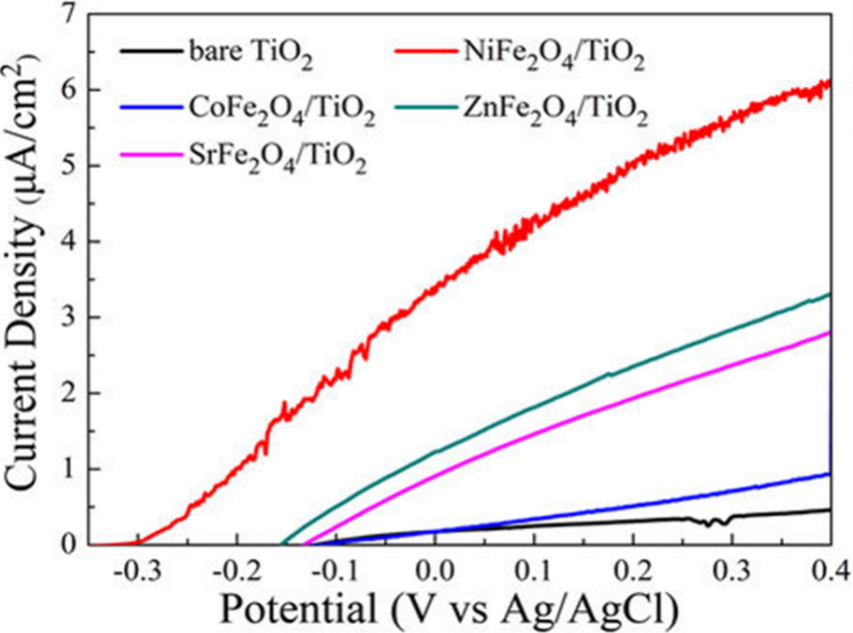
*I*–*V* (current intensity–applied voltage) curve. Reprinted from [97], an article distributed under the Creative Commons Attribution 4.0 license http://creativecommons.org/licenses/by/4.0/ copyright the authors of [97].

**Figure 6:**
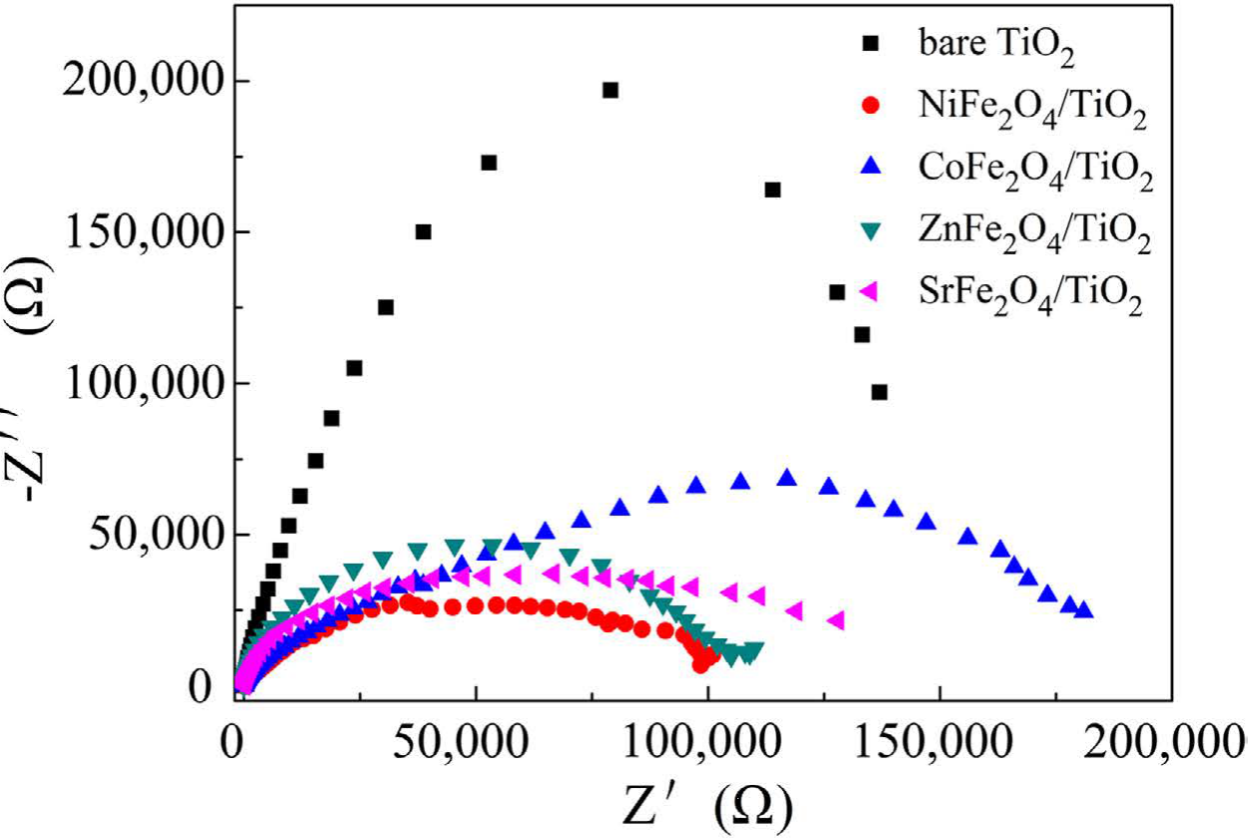
Comparison of arc radius of Nyquist plot between bare TiO_2_ and modified TiO_2_ (MFe_2_O_4_/ TiO_2_) samples (M = Ni^2+^, Co^2+^, Zn^2+^, Sr^2+^). Reprinted from [97], an article distributed under the Creative Commons Attribution 4.0 license, http://creativecommons.org/licenses/by/4.0/, copyright the authors of [97].

The enhanced charge transport efficiency can be found from photoelectrochemical studies using a three electrode system (working electrode, counter electrode and reference electrode). A current intensity–applied voltage (*I*–*V*) curve (Figure 5) obtained from linear sweep voltammetry (LSV) gives the photocurrent generation by an applied bias; the higher the current density, the better the separation of photogenerated charge carriers [97–98]. It has been shown that a more negative open circuit potential (*V*_oc_), results in higher charge carrier separation and transfer [99–101]. Electrochemical impedance studies (EIS) explore the resistance of a material through a Nyquist plot. A smaller arc radius of the Nyquist plot suggests better transfer of charge carriers with lower resistance. The modification of TiO_2_ with ferrites (e.g., MFe_2_O_4_) results in a smaller arc radius of the Nyquist plot, as shown in Figure 6, and hence, better charge transport is observed [97].

#### Modification of TiO_2_ with carbon-based advanced materials

Advanced carbon nanomaterials, such as graphene and its derivatives, carbon nanotubes (CNTs) and carbon dots (CDs), have been used to modify semiconductor photocatalysts in order to promote the separation of photoinduced species and extend the light absorption range, which are crucial for enhanced photoactivity [102–105]. In this section, we have discussed photoreduction of Cr(VI) over TiO_2_ modified with reduced graphene oxides (RGOs), CNTs and CDs. The various preparation methods of modified photocatalysts, conditions for photocatalytic reduction, source of illumination, percentage of Cr(VI) reduction and the superior performance of the composite photocatalysts in comparison with TiO_2_ are listed in Table 1.

**Table 1:** Preparation methods of modified photocatalysts, experimental conditions for photocatalytic reduction of Cr(VI), source of illumination, percentage of Cr(VI) reduction and comparison of the composite modified-photocatalyst performance with TiO_2_. Reduced graphene oxide (RGO), carbon nanotubes (CNTs), carbon dots (CDs), nanorod arrays (NRAs), reduced graphene oxide hydrogel (rGH), TiO_2_ hollow-core–shell microspheres (TGHMs), visible light spectrum (vis), ultraviolet light spectrum (UV).

Photocatalyst	Preparation method	pH	Initial Cr(VI) concentration (mg/L)	Dose (g/L)	Irradiation time (min)	Irradiation source	Cr(VI) reduction (%)	Comparison of performance	Ref.

Carbon-based advanced materials for TiO_2_ modification									

TiO_2_–RGO	microwave assisted reduction	–	10.0	1.0	–	UV	91	1.09 times more than pure TiO_2_; 1.3 times more than P25	[125]
TiO_2_–RGO	sol–gel combustion	2.6	12.0	0.2	240	vis	86.5	1.6 times more than pure TiO_2_	[131]
TiO_2_–rGH	vitamin C assisited sol–gel	5.5	5.0	1.0	30	UV	100	1.6 times more than pure TiO_2_	[132]
TGHMs	hydrothermal etching reaction	–	50.0	1.0	150	vis	50	≈5 times more than pure TiO_2_	[134]
TiO_2_–xRGO	one-step solvothermal	2.0	20.0	0.8	210	vis	96	–	[135]
TiO_2_/CNTs	hydrothermal method	3.0	20.0	1.0	180	UV	67.5	–	[139]
CDs–TNs	hydrothermal method	3.0	10.0	1.0	150	vis	100	≈7 times more than P25	[143]

Semiconductor-oxide-modified TiO_2_									

ZnO–TiO_2_	precipitation	3.0	20.0	1.0	120	UV	99.99	1.16 times more than pure TiO_2_	[92]
ZnO–TiO_2_	wetness impregnation method	5.5	20.0	1.0	–	UV	–	–	[155]
TiO_2_–Fe_3_O_4_	polymerizable sol–gel approach	3.0	7.0	0.3	30	UV	100	–	[158]
WO_3_–TiO_2_ NTs	electrochemical synthesis	2.0	20.0	–	130	vis	100	1.58 times more than TiO_2_ NTs	[159]
Bi_2_O_3_–TiO_2_	sol–gel and hydrothermal methods	3.0	20.0	1.0	180	vis	73.9	reduction by TiO_2_ was negligible	[178]
TiO_2_–Cu_2_O	sol–gel	–	5.0	0.2	90	vis	100	1.8 times more than pure TiO_2_	[96]
NiO–TiO_2_	sol–gel	3.5	9.6	1.0	120	vis	95	1.5 times more than pure TiO_2_	[161]
CuBi_2_O_4_–TiO_2_	nitrate route	4.0	30.0	1.0	<240	sunlight	98	–	[182]
ZnFe_2_O_4_–TiO_2_	nitrate route	3.0	–	1.0	–	vis	–	–	[186]
NiFe_2_O_4_–TiO_2_ NRAs	hydrothermal	–	12.5	–	180	vis	94.18	2.0 times more than pure TiO_2_	[97]
ZnFe_2_O_4_ –TiO_2_ NRAs	hydrothermal	–	12.5	–	180	vis	94.086	2.0 times more than pure TiO_2_	[97]
SrFe_2_O_4_–TiO_2_ NRAs	hydrothermal	–	12.5	–	180	vis	92.39	2.0 times more than pure TiO_2_	[97]

Semiconductor sulfide-modified TiO_2_									

CdS@TiO_2_	two-step solvothermal method	–	–	–	30	vis	100	–	[198]
CdS NSPs@TiO_2_	facile interfacial self-assembly strategy	–	20.0	0.333	40	vis	–	–	[199]
TiO_2_–CdS films	one-step microwave assisted chemical bath deposition method	–	10.0	–	240	vis	93	3 times more than TiO_2_ film	[200]
SnS_2_–TiO_2_	solvothermal reactions	–	–	–	–	vis	100	6.6 times more than pure TiO_2_	[206]

Noble-metal-modified TiO_2_									

Ag–TiO_2_	sol–gel method	2.0	10.0	0.2	240	vis	99.8	–	[217]
Ag–Ag_2_S/TiO_2_	hydrothermal	3.0	10.0	1.0	360	vis	100	3 times more than pure TiO_2_	[219]
Au/N–TiO_2_	modified sol–gel method	–	10.0	1.0	240	vis	90	2.6 times more than pure TiO_2_	[220]
Au/TiO_2_−Pt	–	2.0	103.99	10.0	1440	vis	99	–	[221]
TiO_2_–Au/Pt	–	≈2.5	5.0	0.25	25	UV–vis LED	100	–	[222]
TiO_2_@Au@CeO_2_	hydrothermal route	4.03	5.0	0.3	300	vis	95	2.96 times more than Degussa P25 TiO_2_	[223]
TiO_2_@Pt@CeO_2_	sacrificial template route	–	2.49	0.3	150	vis	99	1.66 times more than TiO_2_	[225]

Dye-sensitized TiO_2_									

(Cu) PP-TiO_2_	–	–	7.06	1.0	400	vis	99	–	[228]
N719 dye–TiO_2_ films	–	2.0	7.06	–	60	vis	99.5	–	[229]

#### Photocatalytic reduction of Cr(VI) over reduced graphene oxide modified TiO_2_

**Figure 7:**
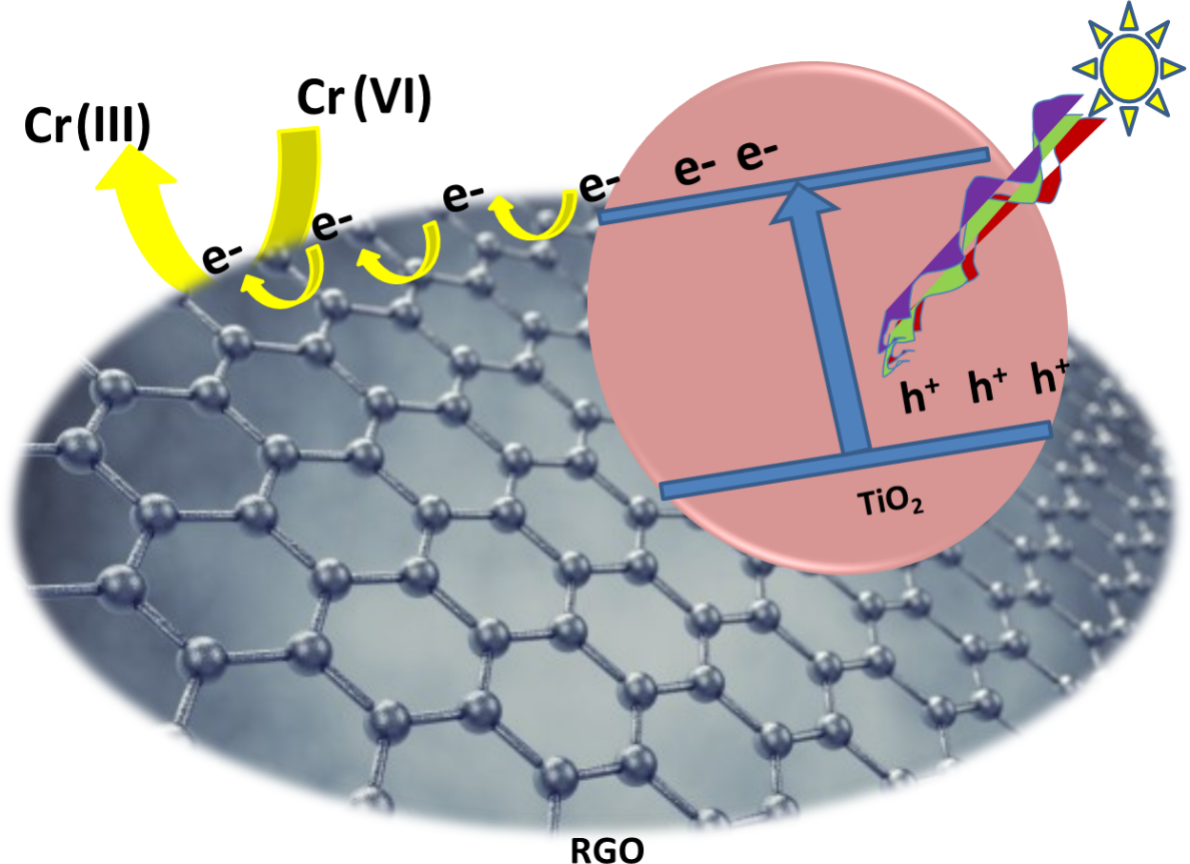
Transport of photoinduced electrons from the conduction band of TiO_2_ through an RGO sheet, resulting in suppression of the recombination of charge carriers, which facilitates enhanced photocatalytic reduction of Cr(VI).

Graphene is a single layer of two-dimensional carbon material with graphite structure. Because of its low cost, excellent conductivity, superior chemical stability and exceptionally high specific surface area, graphene and its derivatives have attracted significant attention for various applications like photocatalysis, energy storage, nano-electronics and photovoltaics [106–110]. In photocatalytic water treatment, these are considered as promising candidates to combine with semiconductors as they have good electron collector and transporter properties. These materials suppress the recombination of charge carriers by effectively transporting the photoinduced electrons of the semiconductor, resulting in high photocatalytic activity [111–116]. In addition to this, graphene support on TiO_2_ results in higher transport of photogenerated charge carriers, enrichment of light harvesting, increase of surface active sites and chemical stability of photocatalysts, which are essentially needed for a good photocatalyst [117–119]. The transport of electrons is facilitated from the semiconductor to graphene only when the work function of graphene is greater than the conduction band energy of the semiconductor. Since the work function of graphene (≈4.42 eV) [120–121] is greater than the conduction band potential of TiO_2_ (−4.21 V vs vacuum) [122–124], photogenerated electrons from TiO_2_ are efficiently transported to graphene, leading to enhanced photocatalytic activity (Figure 7).

Liu et al. reported that TiO_2_–RGO composites exhibited enhanced photocatalytic performance for the reduction of Cr(VI) by UV light illumination as compared to pure TiO_2_ and commercial P25 [125]. The enhancement in the photocatalytic activity is mainly due to two reasons: (i) inhibition in recombination of electron–hole pairs by the effective transport of photoinduced electrons from the CB of TiO_2_ to RGO [126–127], and (ii) higher light absorption due to the development in surface electric charge of the oxides [128]. In addition, the red shift in the absorption edge of the TiO_2_ –RGO composite as compared to pure TiO_2_ is ascribed to the formation of C–O–Ti bonds [126]. It was further observed by Liu et al. that the photocatalytic reduction of Cr(VI) by the TiO_2_ –RGO composite increases with increasing RGO content and reaches a maximum value of 91% for a sample containing 0.8 wt % of RGO. However, upon further increase of the RGO content, the photocatalytic performance deteriorated [125]. This may be due to formation of recombination centers by excess RGO, which facilitates recombination of electron–hole pairs instead of providing an electron pathway [129–130] and maximizes the light harvesting competition between TiO_2_ and RGO [128]. Zhao et al. prepared TiO_2_–RGO composites with large specific surface area (104.9 m^2^/g) [131]. At low pH, the large surface of the composite becomes positively charged, which adsorbed negatively charged Cr(VI) species effectively through electrostatic attraction. This facilitated the photocatalytic reduction of Cr(VI). In addition to this, the grafting of TiO_2_ onto RGO forms C–O–Ti bonds, which extends light absorption of TiO_2_ to longer wavelengths (visible region) for generation of photoelectrons as well as favors the effective transfer of these photoinduced electrons for enhanced photocatalytic activity towards reduction of Cr(VI). About 86.5% of Cr(VI) was photoreduced by TiO_2_–RGO composites, while TiO_2_ photoreduced only 54.2% of Cr(VI). They proposed following mechanism for photocatalytic reduction of Cr(VI) by the TiO_2_–RGO composite. At first, negatively charged Cr(VI) species are bound with the protonated surfaces of TiO_2_–RGO through electrostatic attraction. Then there a photocatalytic reduction of Cr(VI) to Cr(III) occurs under irradiation with visible light, in the second step. Third step involves either release of Cr(III) species into the solution due to their electrostatic repulsion from the protonated surfaces of TiO_2_–RGO or their adsorption by deprotonated surfaces.

Li et al. fabricated a composite of TiO_2_ and reduced graphene oxide hydrogel (rGH). The 3D macrostructures of rGH enhanced the accessible surface area and possessed highly porous structures with a pore size distribution of several micrometers, which enabled the use of the composite for the fast adsorption of Cr(VI) through π–π interactions and a nonporous surface adsorption technique. Moreover, the combination of rGH with TiO_2_ nanospheres suppressed the recombination of photoinduced charges and facilitated the transport of photoelectrons for efficient photocatalytic reduction of Cr(VI) under irradiation with UV radiation. Thus, the fabricated photocatalyst exhibited superior synergetic performance of adsorption and photocatalysis by removing 100% Cr(VI) from a solution containing 5 mg L^−1^ of Cr(VI) within 30 minutes. Under continuous flow conditions, the percentage removal was maintained at 100% till the breakthrough point was achieved [132]. Halloysite–polyaniline core–shell nanotubes exhibited higher Cr(VI) oxyanion reduction and adsorption. The activity could be varied with concentration, pH and dopant acid [133].

**Figure 8:**
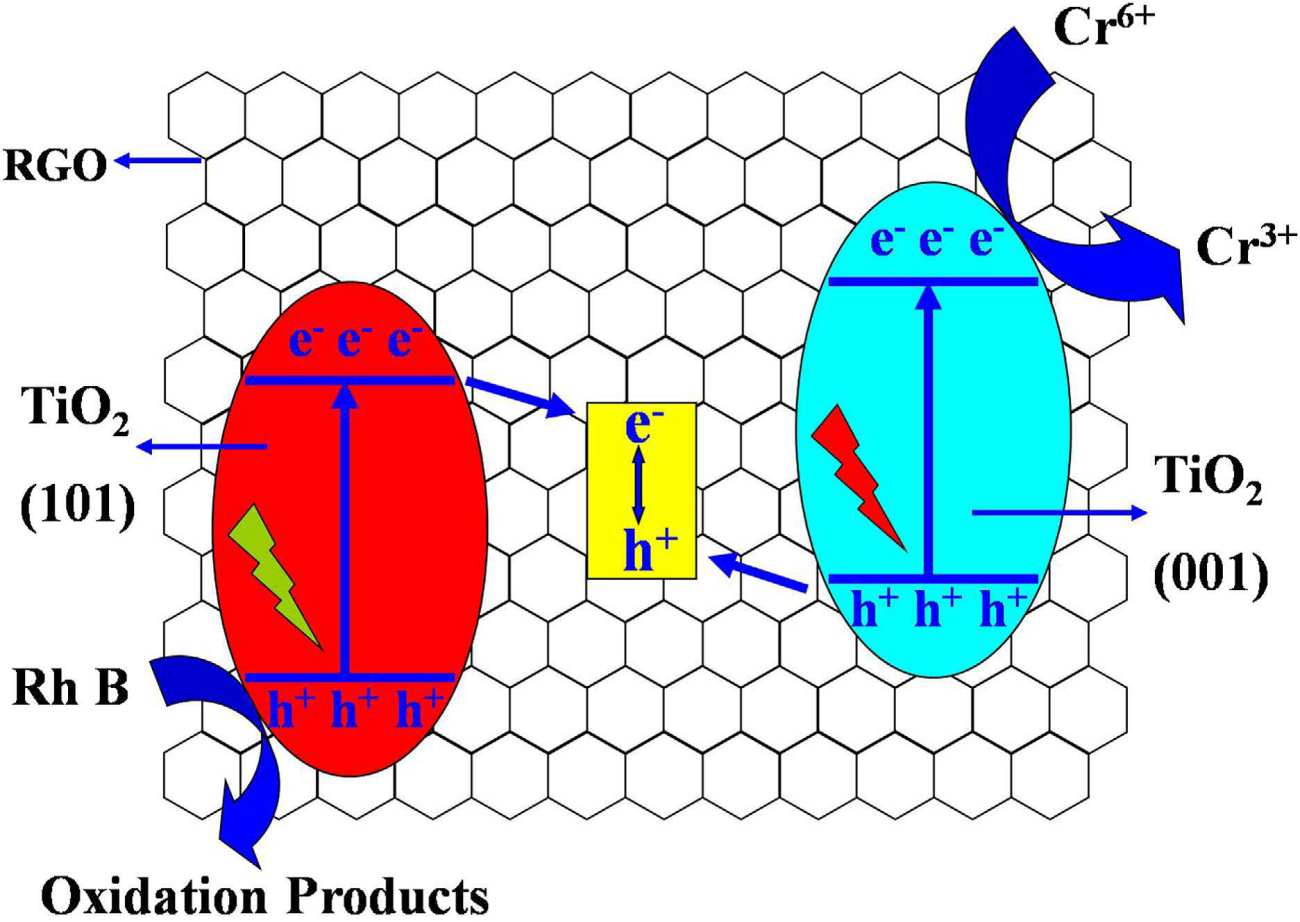
RGO–TiO_2_ core–shell Z scheme for photocatalytic reduction of Cr(VI). Reprinted from [134], an article distributed under the Creative Commons Attribution 4.0 license, http://creativecommons.org/licenses/by/4.0/, copyright the authors of [134].

Graphene-wrapped differently faceted (001 and 101) TiO_2_ hollow-core–shell microspheres (TGHMs) have been fabricated by Liu et al. and were applied for efficient photocatalytic Cr(VI) reduction [134]. They prepared the photocatalyst using a direct-wrapped route followed by hydrothermal etching. The high charge separation efficiency and redox ability are due to the synergetic effect of formation of a Z scheme photocatalytic process and its facilitation by a RGO nanosheets, as shown in Figure 8.

Shaikh et al. synthesized TiO_2_–RGO nanocomposites with uniformly dispersed 4–9 nm diameter TiO_2_ nanoparticles by a one-step solvothermal technique and evaluated its photocatalytic activity towards reduction of Cr(VI). The use of 1 wt % of RGO at acidic pH (pH 2) exhibited higher photoreduction due to the interfacial charge transfer by RGO [135]. A maximum of 96% Cr(VI) reduction was achieved. An RGO–TiO_2_ composite photocatalyst was prepared by a wet impregnation route followed by surface complexation with a simple glucose molecule. The prepared photocatalyst reduced 100% of 30 mg L^−1^ Cr(VI) within 60 minutes under illumination with visible light. The role of glucose is to enhance the light absorption and separation of charge carriers through complex formation with RGO/TiO_2_. This leads to the increase in photocatalytic reduction of Cr(VI) [136].

Chen et al. fabricated Mn-doped TiO_2_–RGO photocatalysts through a one-pot hydrothermal method and studied the photocatalytic reduction of Cr(VI) under solar illumination. The high photocatalytic activity is attributed to the Mn doping and synergetic effect of adsorption and photocatalysis by the RGO support. The photogenerated electrons are transported from the Mn-doped TiO_2_ through RGO and were found to reduce the adsorbed Cr(VI) [137]. A Ti–SBA-15–g-C_3_N_4_ material was also shown to exhibit higher Cr(VI) photoreduction under visible light illumination. The Ti moiety in Ti–SBA-15 can extract the conduction band electrons of g-C_3_N_4_ after visible light irradiation followed by transfer of electrons to Cr(VI) to produce Cr(III) [138].

#### Photocatalytic reduction of Cr(VI) using CNT-modified TiO_2_

Carbon nanotubes (CNTs) have also been shown to possess excellent electronic and conductive properties. Waldmann et al. studied photocatalytic reduction of Cr(VI) over TiO_2_-coupled CNTs. The reduction rate of Cr(VI) increased due to transfer of photogenerated electrons through CNT surfaces in the absence of sacrificial agents [139]. Huang et al. reported that a simultaneous photocatalytic degradation of Cr(VI) and phenol occurs over CNT-modified TiO_2_.The high photoactivity of CNT–TiO_2_ may be attributed to the synergistic effect of adsorption and electron trap properties of the CNTs [140].

#### Photocatalytic reduction of Cr(VI) using carbon-dot-modified TiO_2_

Carbons dots (CDs) are now being widely investigated as co-catalysts because of their intriguing properties such as small size, high dispersion, abundant surface functional groups, unique photoluminescence and good electron transfer ability [141–142]. Carbon dot–TiO_2_ (CD–TiO_2_) nanosheet composites synthesized by a hydrothermal route were studied for photoreduction of Cr(VI) under sunlight illumination [143]. Its enhanced photoreduction capacity over TiO_2_ nanosheets, P25 and CD–P25 was attributed to the better charge transfer as well as higher light absorption properties of CDs. The in situ formation of H_2_O_2_ promotes the photoactivity to a great extent. Zhang et al. synthesized CDs coupled with TiO_2_ mesocrystals (CD/MT) in which the CDs acted as both the electron collectors and the active sites [144]. The negatively charged Cr(VI) species adsorbed effectively onto the positively charged surface of the CD/MT followed by photoreduction of Cr(VI) to Cr(III) ions that could be desorbed easily from the surface. Therefore, the selective adsorption–desorption phenomena facilitated the recycling ability of CD/MT and enhanced its photoreduction efficiency by 5.4 fold as compared to that of TiO_2_.

#### Modifications of TiO_2_ with semiconductor oxides for photocatalytic reduction of Cr(VI)

Modification of TiO_2_ with semiconductor oxides having a suitable band gap is a novel approach for significant charge separation, long lifetime of the charge carriers and effective interfacial charge transfer, which are properties that lead to enhanced photocatalytic activity. This also enhances the light absorption range towards longer wavelengths. The band edge potentials and band gaps of different semiconductor oxides and sulfides are given in Figure 2.

#### Photocatalytic reduction of Cr(VI) over TiO_2_ modified with simple transition metal oxides under UV irradiation

Transition metal oxides such as ZnO have been combined with TiO_2_ to form composite photocatalysts, which are used efficiently for photocatalytic reduction of Cr(VI). ZnO has been recognized as a potential photocatalyst for extensive environmental applications because of its availability and low cost. It also possesses intriguing optical and electric properties [145–148]. Studies involving ZnO-mediated photoreduction of Cr(VI) have been carried out under illumination with UV radiation [149]. Since the conduction band edge potential for TiO_2_ is more positive than that of ZnO (Figure 2), the combination of ZnO with TiO_2_ can cause transfer of electrons from the CB of ZnO to that of TiO_2_ and holes from the VB of TiO_2_ to that of ZnO, upon UV irradiation. This leads to effective separation of photoinduced charge carriers, which was shown to enhance the photocatalytic activity of a ZnO/TiO_2_ composite [150]. Hence, ZnO is considered as a suitable semiconductor to be coupled with TiO_2_ [151–153].

Joubani and coworkers reported that a ZnO/TiO_2_ composite photocatalysts exhibited superior photocatalytic performance by reducing a maximum of 99.99% of Cr(VI) as compared to TiO_2_ and ZnO, which reduced 86.07% and 82.33% of Cr(VI), respectively. Its better performance was also evident from the consumption of the lowest electrical energy per order of magnitude for photocatalytic reduction of Cr(VI) as compared to that in UV/ZnO and UV/TiO_2_ systems [92]. The rate of photocatalytic reduction of Cr(VI) was increased by increasing the photocatalyst dose [154]. Ku et al. reported that the combination of ZnO on the surface of TiO_2_ at a higher calcination temperature (>500 °C) prevents the transformation of anatase to rutile phase. It also enhances the specific surface area of the ZnO/TiO_2_ composite by inhibiting aggregation and agglomeration of particles. On increasing ZnO content in the ZnO/TiO_2_ composite, the rate of reduction of Cr(VI) was increased and the sample containing 2.0 mol % ZnO exhibited a maximum photocatalytic reduction of Cr(VI) in aqueous solution as the recombination of charge carriers is suppressed by the effective transfer of electrons from ZnO to TiO_2_ [155]. Further increase in ZnO content created new recombination centers of electron–hole pairs by abundantly available ZnO particles, resulting in a decrease in the degradation rate [156]. The photocatalytic reduction of Cr(VI) was decreased with further increase in the calcination temperature because of the decrease in the specific surface area induced by the aggregation and agglomeration of particles [157].

TiO_2_/Fe_3_O_4_ composite photocatalysts were synthesized through a polymerizable sol−gel route to investigate the photocatalytic reduction of Cr(VI) under UV light irradiation. The anchoring of TiO_2_ over Fe_3_O_4_ resulted in (i) high dispersion of the active site, which is important for achieving higher reaction rate, (ii) enhancement of the photoreduction rate by decreasing the recombination of electron−hole pairs due to significant overlap of the TiO_2_ band with that of Fe_3_O_4_ and (iii) efficient separation and recyclability of the catalyst under application of an external magnetic field because of the presence of magnetic Fe_3_O_4_. Therefore, the composite photocatalysts exhibited a higher rate of photoreduction of Cr(VI) as compared to the nonsupported bulk TiO_2_ as well as calcined Fe_3_O_4_. In fact, 30% TiO_2_/Fe_3_O_4_ has shown the highest Cr(VI) photoreduction rate due to formation of effective heterojunction by the loading of 30% TiO_2_ over Fe_3_O_4_ [158].

#### Visible-light responsive, transition metal oxide modified TiO_2_ for photocatalytic reduction of Cr(VI)

Two types of transition metal oxides have been combined with TiO_2_ for photocatalytic reduction of Cr(VI). One type are simple metal oxides with the common formula MO*_x_* and the other are mixed metal oxides of general formula MM′O*_x_*, where M and M′ represent transition metal ions and *x* represents an integer.

**Figure 9:**
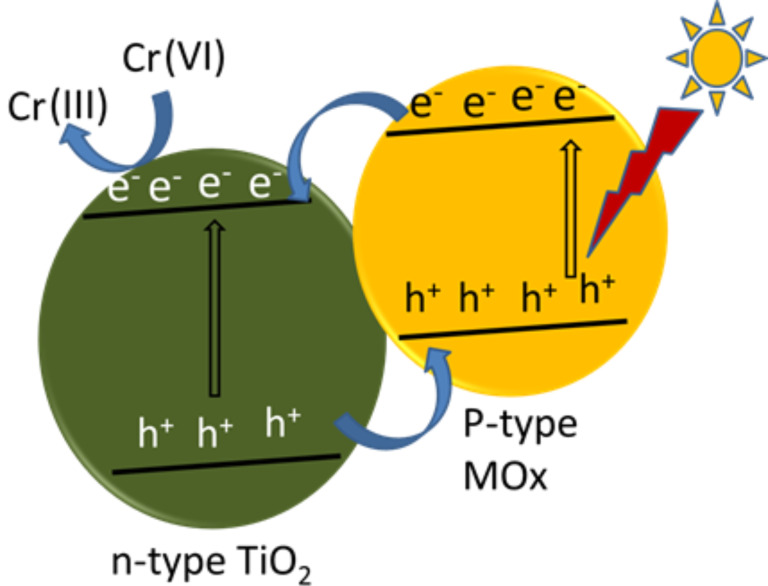
Mechanism for photocatalytic reduction of Cr(VI) by TiO_2_–MO*_x_* under irradiation of visible light.

**Photocatalytic reduction of Cr(VI) by TiO_2 _modified with simple metal oxides:** The coupling of TiO_2_ with simple metal oxides such as Bi_2_O_3_, WO_3_, or Cu_2_O is a promising strategy to design highly efficient photocatalysts [86,159–160]. The recombination of the photogenerated charge carriers is not only suppressed but also the spectral response of TiO_2_ is extended to the visible spectrum by combining these metal oxides [159,161]. Moreover, formation of a p–n heterojunction is another strategy to facilitate the effective separation of electron–hole pairs and to extend light absorption to the red end of the solar spectrum [162–164]. The p–n junction is formed by coupling a p-type (hole-rich) semiconductor with n-type (electron-rich) TiO_2_. As a result, the photoelectrons are diffused to the p-type semiconductor and holes are diffused to n-type TiO_2_ to create an inner electric field at the interface of electron–hole diffusion. The inner electric field thus formed acts as a potential barrier to inhibit the recombination of these charge carriers by escalating the transport of electrons from p-type to n-type and that of holes from n-type to p-type semiconductors. Several studies have been reported on photocatalytic reduction of Cr(VI) by TiO_2_–p-type semiconductor metal oxide heterojunctions. The mechanism for visible-light-driven photocatalytic reduction of Cr(VI) on TiO_2_–p-type metal oxide heterojunctions is explained in Figure 9.

Cuprous oxide (Cu_2_O) is a p-type semiconductor having band gap energy of 2.17 eV. It possesses a high absorption coefficient over the whole visible region, and hence, is used as a suitable solar energy converter [160]. Moreover, it is nontoxic and highly abundant in the earth’s crust [163]. Abdullah and co-investigators deposited p-type Cu_2_O on TiO_2_ nanoparticles to form TiO_2_/Cu_2_O nanocomposites, a p–n nano-heterojunction having a built-in electric field at the interfaces. This built-in electric field largely prevented the recombination of photoexcited charge carriers, resulting in increased lifetime of photocarriers, induced higher quantum efficiency and a largely enhanced photocatalytic performance. It also caused a drift of photogenerated electrons to the CB of TiO_2_ for reduction of Cr(VI) to Cr(III) and that of holes to the VB of Cu_2_O for oxidation of water to oxygen under visible-light irradiation. Since green colored precipitates of Cr(OH)_3_ are formed on the surfaces of TiO_2_/Cu_2_O nanocomposites, the possibility of re-oxidation of Cr(III) to Cr(VI) was avoided although the reduction potential of Cr(III) was more negative than that of water [96]. The oxidation of water to O_2_ was confirmed from the photocatalytic oxidation of water [165]. Upon increasing the content of Cu_2_O in TiO_2_/Cu_2_O nanocomposites, photoreduction increased and reached a maximum for 30% Cu_2_O. This is because 30% Cu_2_O might be an appropriate amount for the formation of a p–n junction between TiO_2_ and Cu_2_O nanoparticles, which could efficiently separate photogenerated charge carriers for higher photoactivity under visible light. Almost 100% of a 10 ppm K_2_Cr_2_O_7_ solution was degraded in 90 min. A further increase in the concentration of Cu_2_O would cover the surface of TiO_2_ and retard the transfer of photoelectrons to outer surface. The percentage photoreduction of Cr(VI) decreases with the increase in number of cycles, mainly due to deposition of Cr(OH)_3_ on the surfaces of the nanocomposites. Cu_2_O with several crystal structures such as octahedrons, rhombic, dodecahedrons and cubes with different facets were synthesized and their efficiency towards photocatalytic reduction of Cr(VI) was investigated by Qin et al. [166]. Zhong and co-workers fabricated Cu-decorated TiO_2_ nanotube photoelectrodes by a facile hydrothermal method. The optoelectronic coupling between Cu nanoparticles and TiO_2_ nanotubes enhanced the rate of transfer of electrons and subsequently suppressed the electron/hole pair recombination due to which photoreduction of Cr(VI) was increased [167]. Cu/Cu_2_O-decorated TiO_2_/alginate beads synthesized by a novel, environmentally friendly polyol process exhibited excellent photoreduction of Cr(VI). The superior performance may be attributed to the homogeneous TiO_2_ dispersion, presence of Cu nanoparticles for facilitating charge separation processes, synergetic effect of the TiO_2_/Cu_2_O heterojunction and the small size of the photocatalyst [168].

Velegraki et al. fabricated 3D mesoporous networks of assembled CoO nanoparticles (CoO MNAs) to study its photocatalytic behavior towards reduction of Cr(VI) under UV and visible light irradiation. The enhanced photocatalytic performance may be attributed to its accessible pore volume, appropriate band edge positions and specific reactivity of the crystal phase. Photocatalytic reduction of Cr(VI) proceeds with simultaneous formation of molecular oxygen and hydroxyl radicals at the CoO surface [169]. NiO is a p-type semiconductor, which can conveniently be combined with different photocatalysts and the composite photocatalysts exhibit higher activity [170–172]. The introduction of nickel oxide into the TiO_2_ matrix restricts the transformation of anatase to rutile phase possibly because of the presence of Ni^2+^ ions that stabilize the anatase phase. Further, the presence of NiO would hinder the aggregation of TiO_2_ particles, resulting in increase of surface area and decrease of particle size of the photocatalyst [173]. An increase in the surface area of NiO/TiO_2_ will lead to an increase of active sites, which enhances the photocatalytic activity. Ku et al. reported that coupling of p-type NiO with n-type TiO_2_ resulted in the development of an NiO/TiO_2_ photocatalyst with the formation of a p–n junction [161]. The inner electric field developed by the thus formed p–n junction separated the photogenerated holes and electrons effectively by transferring the holes into NiO and electrons into TiO_2_. As a result, the coupled photocatalyst exhibited a higher photovoltage intensity and enhanced photocatalytic activity towards reduction of Cr(VI). However, the photoactivity was reduced with increasing NiO dosage because excess NiO acted as the recombination centers for photogenerated charge carriers. Therefore, a photocatalyst containing 0.1% NiO and calcined at 500 °C exhibited maximum Cr(VI) reduction. In addition to this, the introduction of NiO resulted in good contact between NiO and TiO_2_, and as a consequence, the Ti 3d and Ni 3d sates are overlapped to form a modified conduction band. This caused band gap reduction resulting in a red shift of the absorption. Hence, the coupling of NiO with TiO_2_ not only retards the recombination of photogenerated electrons and holes, but also extends the absorption edge towards the visible region. Moreover, an increase in the Ni content hinders aggregation of TiO_2_ because an appreciable amount of NiO restricts the growth of TiO_2_ particles. The inhibition in aggregation resulted in an increase of the surface area, which improves the active sites that promote the photocatalytic activity of NiO/TiO_2_ particles [161].

Since the ionic radius of W^6+^ is similar to that of Ti^4+^, coupling of TiO_2_ with WO_3_ forms a well-doped WO_3_/TiO_2_ composite, which can be applied for photocatalytic degradation of pollutants under visible light irradiation. Yang et al. reported that the photoreduction of Cr(VI) by WO_3_-doped TiO_2_ nanotube (NT) arrays was found to be greater than that of neat TiO_2_ NT arrays [159]. This is because the incorporation of WO_3_ with TiO_2_ facilitates the separation of photoinduced charge carriers and shifts the absorption edge to the visible region by reducing the band gap of TiO_2_ [162,174]. The highest photoreduction efficiency of Cr(VI) was obtained with WO_3_/TiO_2_ NTs containing 1% tungsten (W) as it provides highest photocurrent and creates photogenerated carriers with the longest lifetime. On further increasing the W content, photocurrents are decreased because excess WO_3_ nanoparticles serve as the recombination centers. Moreover, the decrease of the interfacial charge space of the WO_3_/TiO_2_ NTs with increasing W content was also responsible for the reduction of photocurrent. Dozzi et al. synthesized a series of titanium–tungsten mixed oxides through coupling of TiO_2_ with varying WO_3_ percentage by a base-catalyzed sol–gel method [175]. WO_3_ plays a vital role in inhibiting charge recombination for efficient charge transfer to enhance the Cr(VI) reduction.

Bi_2_O_3_ possesses a narrow band gap (2.8 eV), appropriate valence band position, and similar photocatalytic mechanism to that of TiO_2_. Hence, it can conveniently be coupled with TiO_2_ [176–177]. Yang and co-workers prepared Bi_2_O_3_/TiO_2_ coupled photocatalysts by a sol–gel method followed by a hydrothermal technique. The coupling of Bi_2_O_3_ not only hindered the transformation of anatase phase to rutile but also facilitated the extension of the absorption range to the visible region. It also escalated the interfacial charge transfer between Bi_2_O_3_ and TiO_2_. The maximum photocatalytic activity under irradiation of visible light for reduction of Cr(VI) was exhibited by 2.0% Bi_2_O_3_/TiO_2_. A further increase in Bi_2_O_3_ dosage may create new recombination centers of photoinduced charge carriers, which in turn decreased the photocatalytic activity [178].

**Photocatalytic reduction of Cr(VI) by TiO_2 _modified with spinel metal oxides:** Spinel types of metal oxides with the general formula AB_2_O_4_ (where A is a divalent metal ion and B is a trivalent metal ion) possess narrow band gaps which enable them to absorb throughout the visible region [179]. In addition, these materials have a high tendency for conduction of electrons because the hopping of electrons takes place between different valence states of metals in O-sites. This caused efficient transfer of charge carriers [180]. Hence, spinel oxides are being recognized as the potential photocatalysts.

The modification of TiO_2_ with these metal oxides has shown promising behavior in photocatalytic Cr(VI) reduction. Gherbi et al. reported the visible-light-driven photoreduction of Cr(VI) over CuAl_2_O_4_/TiO_2_ [181] with 95% reduction after 3 h irradiation at pH 2. The photoreduction follows first order kinetics with a half-life of ≈1 h and a quantum yield of 0.11%. Photocatalytic reduction of chromate ions under sunlight over CuBi_2_O_4_/TiO_2_ has also been reported by Lahamar et al. A remarkable performance of 98% reduction is obtained in less than 4 h for a Cr(VI) concentration of 30 mg L^−1^ at pH ≈4 by using 1 g L^−1^ catalyst. The kinetics of chromate photoreduction is well described by the Langmuir–Hinshelwood model [182]. The heterosystem CuCo_2_O_4_/TiO_2_ for the removal of Cr(VI) by photocatalytic reduction under visible light has been reported by Kebir et al. [183]. The synergetic effect of adsorption and photocatalytic reduction with proper band alignment are attributed to enhanced Cr(VI) removal from tannery wastewater.

Transition metal ferrites have also been combined with TiO_2_ for photocatalytic reduction of Cr(VI) not only due to their efficient visible-light-induced photocatalytic activity, but also due to their high photostability, good super-paramagnetic behavior, nontoxicity, facile fabrication, enhanced adsorption ability, low cost and abundant availability [184–185]. The Trari group also reported photocatalytic reduction of Cr(VI) by spinel ZnFe_2_O_4_. The photoelectrons generated in ZnFe_2_O_4_ are injected into TiO_2_ and subsequently transferred to Cr(VI), which is reduced to a trivalent state [186]. Gao et al. fabricated MFe_2_O_4_ (M = Ni^2+^, Zn^2+^, Co^2+^ and Sr^2+^) modified TiO_2_ nanorod arrays (NRAs) to compare their photoelectrochemical and photocatalytic activity with that of bare TiO_2_ NRAs towards reduction of Cr(VI) [97]. All the modified TiO_2_ NRAs exhibited strong visible light absorption due to the intrinsic band gap absorption of MFe_2_O_4_. Since the CB of MFe_2_O_4_ is more positive than that of TiO_2_, the excited electrons can move from MFe_2_O_4_ to the CB of TiO_2_, whereas the generated holes are accumulated in the VB of MFe_2_O_4_. This leads to an effective charge transfer, leading to longer lifetime. As a result, NiFe_2_O_4_/TiO_2_ NRAs, ZnFe_2_O_4_/TiO_2_ NRAs and SrFe_2_O_4_/TiO_2_ NRAs exhibited enhanced photocatalytic activity as compared to bare TiO_2_ NRAs. On the other hand, the CB of CoFe_2_O_4_ is more positive than that of TiO_2_, while its VB is more negative than that of TiO_2_ [50,187]. This made the CoFe_2_O_4_/TiO_2_ heterojunction nonconductive, resulting in inefficient separation of photoexcited charge carriers and hence poor photocatalytic activity was achieved.

#### Metal-sulfide-modified TiO_2_ as visible-light-responsive photocatalysts for photoreduction of Cr(VI)

Metal sulfides such as CdS and SnS_2_ are considered as potential candidates for harvesting light in the visible region due to their narrow band gap and are being used as visible-light-responsive photocatalysts in wastewater treatment for degradation of pollutants [188–191]. These can also act as promising sensitizers for wide band gap semiconductors such as TiO_2_ [192]. In this section, we discuss the photocatalytic activity of metal sulfide modified TiO_2_ towards reduction of Cr(VI).

Cadmium sulfide (CdS) is an important semiconductor with a direct band gap of 2.4 eV that corresponds well with the visible region of the electromagnetic spectrum. Thus, it is considered as an excellent visible-light induced photocatalyst. Moreover, it has a more negative conduction band edge potential with respect to H^+^/H_2_ redox potential. However, its application is greatly limited in photocatalysis due to the very fast rate of recombination of photogenerated charge carriers and high photocorrosion affinity in the presence of solar light [193–195]. The coupling of CdS with another semiconductor is a suitable strategy to overcome these restrictions [196]. When CdS is loaded onto TiO_2_, the surrounding matrix of the later prevents the former from photocorroding [197]. In addition, CdS acts as a photosensitizer to absorb visible radiation and transfers e_CB_^−^ to the CB of TiO_2_ by retaining h_VB_^+^ at its VB. As a result, the recombination of photoinduced species is appreciably inhibited [70]. Therefore, it is often combined with TiO_2_ for enhanced photocatalytic reduction of Cr(VI). A one-dimensional CdS–TiO_2_ core–shell (CdS@TiO_2_) nano-photocatalyst possessed higher reduction and selectivity of Cr(VI) due to the core–shell structure where h_VB_^+^ are trapped by the TiO_2_ shell [198]. Ultrathin TiO_2_-coated CdS core–shell spheres have also been prepared by Chen et al. A coating of an ultrathin TiO_2_ layer on CdS nanoparticles imparts good light harvesting properties, enhanced adsorption capacity, effective charge transport and longer lifetime of excitons, for which the core–shell spheres exhibited higher efficiency for photoreduction of Cr(VI) [199]. Liu et al. reported that CdS sensitization can enhance the photocatalytic performance of TiO_2_ films with a maximum reduction rate of 93% for 240 min under white LED light irradiation as compared to that of pure TiO_2_ film (31%). This was attributed to an increase in light absorption and reduction in the recombination of injected electrons from CdS to TiO_2_ [200].

SnS_2_ is a p-type semiconductor with a band gap of 2.2 eV, which is suitable for visible light absorption (λ > 420 nm). It is harmless, chemically stable and of low cost [201]. It also exhibits relatively higher stability against oxidation and photocorrosion as compared to CdS. Hence, SnS_2_ is considered as a promising photocatalyst among the semiconductor metal sulfides [202–203]. Mondal et al. have synthesized shape oriented SnS_2_ nanostructures by a facile fabrication route on a large scale [204]. The nanoyarn and nanoflower materials were investigated for photoreduction of Cr(VI) under visible light. The enhanced photoactivity of nanoflowers compared to nanoyarn is attributed to a higher surface area and higher photoabsorption. Qu et al. fabricated a corallite-like nanocomposite by surface modification of SnS_2_ and spirobenzopyran derivative (SPNH) with macroporous ordered siliceous foam (MOSF). SnS_2_ nanocrystals exhibited enhanced photocatalytic reduction of Cr(VI) under visible light irradiation after being encapsulated into the matrix of MOSF. On the other hand, SPNH decorated on the surface of MOSF generated phenoxy groups by a ring opening reaction in the presence of UV light. The phenoxy groups thus formed could chelate soluble Cr(III) selectively through ligand coordination. As a result, the corallite-like nanocomposite detoxified Cr(VI) from the contaminated solution through visible-light-induced photocatalysis followed by adsorption of Cr(III). Furthermore, the photocatalyst is stable after three cycles of Cr(VI) degradation [205]. A heterojunction structure of SnS_2_/TiO_2_ nanocomposites was prepared by Zhang et al. [206]. Both VB and CB potentials of SnS_2_ are more negative than those of TiO_2_ due to which photogenerated electrons are transferred efficiently from the CB of SnS_2_ to that of TiO_2_ under irradiation of visible light, and the photogenerated holes remain on the VB of SnS_2_ [207]. This leads to effective separation of photogenerated electrons and holes in SnS_2_. Therefore, the lifetime of the charge carriers is increased owing to interfacial charge transfer to the adsorbed substrates [208–210]. Moreover, TiO_2_ can be sensitized due to this electron transfer process. As a result, the photogenerated electrons reduced Cr_2_O_7_^2−^ to Cr(III) and the holes oxidized water to O_2_ in the absence of extra reducing agents or hole scavengers [211–213]. Hence, the nanocomposite photocatalyst (SnS_2_/TiO_2_) exhibited higher visible-light-driven photocatalytic activity in reduction of Cr(VI) as compared to individual SnS_2_ and TiO_2_. Furthermore, the composition of the composite plays an important role in obtaining high photocatalytic efficiency. When the TiO_2_ content is less, the surfaces of SnS_2_ nanoparticles are insufficiently covered with TiO_2_ nanoparticles, resulting in inhibition of interfacial electron transfer from SnS_2_ to TiO_2_. This leads to poor photocatalytic activity. On the other hand, excess TiO_2_ on the SnS_2_ surface possibly blocked the light irradiation on SnS_2_ and hindered the contact of SnS_2_ with aqueous Cr(VI), due to which the rate of photoreduction is decreased. Therefore, the SnS_2_/TiO_2_ nanocomposite containing an adequate amount of TiO_2_ (44.5%) possessed the highest photocatalytic activity for reduction of Cr(VI). It also exhibited higher photocatalytic activity in comparison with the materials prepared by simple physical mixing of SnS_2_ and TiO_2_ nanoparticles with the same composition. This suggested that SnS_2_ and TiO_2_ nanoparticles were well-mixed and closely contacted with one another in the composite. As a result, the composite provided a better heterojunction interface for effective charge transfer and decreased self-agglomeration [206]. Similar observations were obtained for SnS_2_/TiO_2_ nanocomposites prepared by depositing smaller nanocrystals of TiO_2_ on the surface of larger SnS_2_ nanocrystals [93].

#### Photocatalytic reduction of Cr(VI) using noble-metal-modified TiO_2_

**Figure 10:**
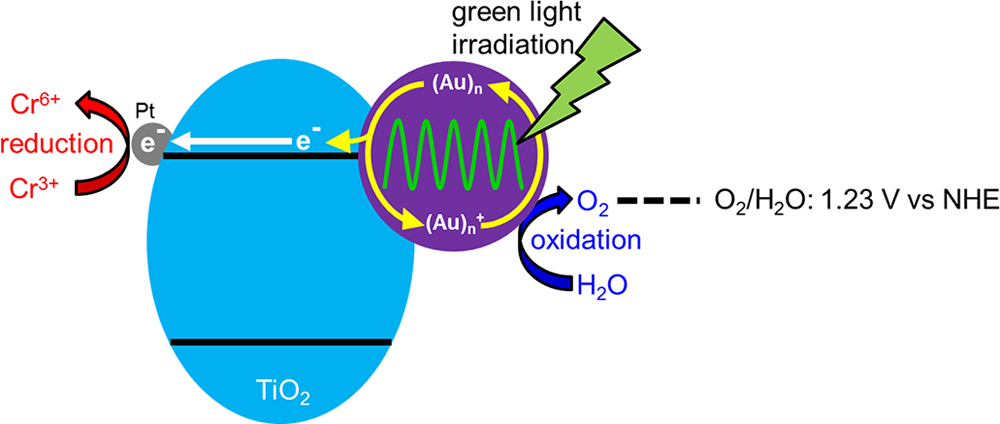
Mechanism of reduction of Cr(VI) using a Au/TiO_2_−Pt plasmonic photocatalyst under visible-light irradiation. Reprinted with permission from [221], copyright 2013 American Chemical Society.

The modification of TiO_2_ by noble metals such as Ag, Au, Pt and Pd facilitates photocatalytic activity due to the significant visible light absorption ability and prominent efficiency in separation of photogenerated charge carriers of these metals. Ag-loaded TiO_2_ photocatalysts were prepared by Liu et al. through the photochemical impregnation method for photocatalytic reduction of Cr(VI) and the enhanced activity was attributed to the co-catalytic behavior of Ag and Ti^3+^ species formed after Ag modification [214]. Noble metal (Ag, Pd, Pt) deposited TiO_2_ with oxygen vacancies were fabricated by Pan and Xu for visible-light-active photocatalytic reduction of Cr(VI). The deposition of noble metal can effectively facilitate the charge transfer efficiency of TiO_2_ and oxygen vacancy creation enhances the light absorption [215]. Magnesium and silver co-impregnated TiO_2_ nanoparticles were prepared by Eskandarloo et al. for photoreduction of Cr(VI) [216]. Lei et al. reported that the surface plasmon absorption of spatially confined electrons in Ag nanoparticles extends the light absorption of Ag-doped TiO_2_ nanomaterials to the visible region. Furthermore, the presence of Ag ions inhibits the recombination of photoinduced species in TiO_2_. As a result, Ag–TiO_2_ exhibited enhanced visible light induced photoactivity towards reduction of Cr(VI) [217]. Co_3_O_4_/Ag/TiO_2_ nanotubes arrays synthesized via photodeposition of Ag and modification of Co_3_O_4_ for enhancement in visible-light photoelectrochemical performance have been studied by Zhang et al. [218]. Photoreduced Ag acted as a bridge that transferred the electrons from Co_3_O_4_ to TiO_2_ for simultaneous Cr(VI) reduction and pollutant oxidation. Hollow spherical Ag–Ag_2_S–TiO_2_ was prepared through in situ chemical transformation of sacrificial Cu_2_S templates with AgNO_3_ solution [219]. The enhanced photoreduction of Cr(VI) is attributed to the synergetic effect of the heterojunction and Schottky barrier that transfer the photogenerated electrons more efficiently. The introduction of Au facilitates the transfer of photogenerated electrons from the CB of TiO_2_ to the Au surface, resulting in the effective separation of charge carriers and easy availability of electrons for Cr(VI) reduction. It was also reported that about 90% Cr(VI) reduction was achieved by the photocatalyst containing 0.3 wt % Au. Further increases in Au content decreased the photoreduction because an excess amount of Au can create recombination centers for electron–hole pairs and can cause a light harvesting competition between N-TiO_2_ and Au [220]. Tanaka et al. investigated the reduction of Cr(VI) over the functionalized plasmonic photocatalyst Au/TiO_2_−Pt under irradiation with visible light. The mechanism of photoreduction is explained in the Figure 10. Owing to the surface plasmon resonance (SPR) phenomenon, Au nanoparticles absorb photons from visible light and release electrons. These electrons are transferred from Au to the Pt co-catalyst through the CB of TiO_2_, since the Fermi level of Pt is lower than that of Au. The photogenerated electrons on Pt reduce Cr(VI) to Cr(III). In the meantime, electron-deficient Au particles are converted to their original metallic state by oxidizing H_2_O to O_2_ [221].

The role of Au and Pt on the photocatalytic activity of anatase TiO_2_ nanosheets of {001} through {101} surface heterojunction for reduction of Cr(VI) was explained by Wang et al. [222]. The conduction power of TiO_2_ was greatly increased on excitation with a UV LED. Au nanoparticles deposited on the {101} facet produced hot electrons in the presence of green LED illumination due to the SPR effect. These hot electrons are transported to the Pt surface through the {101} facet, resulting in effective separation of electron–hole pairs. The photoelectrons at the surface of Pt reduced Cr(VI) to Cr(III). Overall, the {001} through {101} surface heterojunction, effective excitation of TiO_2_ and the synergistic effects of selectively deposited Au and Pt significantly improved the photocatalytic reduction of Cr(VI). A TiO_2_–CeO_2_ multilayer-shell-based core–shell photocatalyst was prepared by Cai et al. through a hydrothermal route using polystyrene as a template, and its photocatalytic activity was evaluated [223]. Au loading into TiO_2_–CeO_2_ core–shell nanostructures enhanced the photoactivity owing to the sandwich nanostructure of multishells of both the oxides and Au as a co-catalyst. A similar observation has also been reported by Pandikumar et al. in the case of silicate-supported Au–TiO_2_ nanotubes, where the role of Au is to enhance the charge transport by acting as a co-catalyst [224]. Li et al. studied the Pt@TiO_2_@CeO_2_ system for Cr(VI) photoreduction, where Pt acts as co-catalyst for better charge transport [225].

#### Dye-sensitized TiO_2_ photocatalysts for Cr(VI) reduction

**Figure 11:**
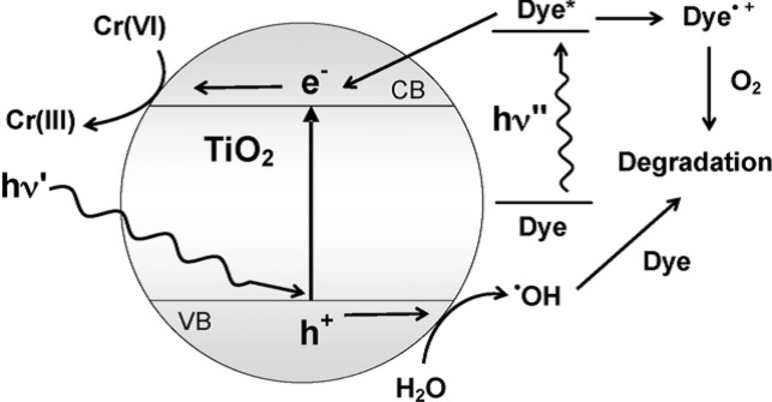
Mechanism for the photocatalytic Cr(VI) reduction by a dye-sensitized TiO_2_ nanocatalyst.

To enrich the light harvesting properties of wide band gap semiconductors, dye sensitization is a useful technique and gained huge attention after the discovery of Gratzel’s dye-sensitized solar cell. The mechanism involves the excitation of dye molecules in the visible range and then charge transfer to the surface of the semiconductor. Methylene blue, erythrosin B, thioine and xanthane are some of the dyes which are commonly used in the sensitization process [226]. The Selli group have studied the photocatalytic reduction of Cr(VI) by taking dye-sensitized Au-deposited TiO_2_. The mechanism of the dye-sensitized TiO_2_ photocatalysis for Cr(VI) reduction is illustrated in Figure 11 [227]. The extra absorption bands of porphyrin dye make it a potential dye-sensitized visible-light-active material for photocatalytic applications. Kar et al. have loaded copper (II) ion in a protoporphyrin IX–TiO_2_ microsphere mixture and studied the effective photoreduction of Cr(VI) under visible light [228].

Cr(VI) photoreduction by a TiO_2_ film and a platinum anode was investigated by Wu et al. [229]. The TiO_2_ film consists of two zones: a dye-sensitized zone and a catalysis zone. In the dye-sensitized zone, light absorption and charge separation are accomplished, while in the catalysis zone, the electrons convert Cr(VI) to Cr(III). Copper azomethyn-bridged phenolic phthalocyanine dye functionalized on TiO_2_ was investigated for visible-light-active Cr(VI) reduction by Albay et al. [230] and an enhanced performance was observed in the presence of nanometer-sized TiO_2_.

A highly effective iron metal-framework photocatalyst (MIL-68(Fe)) has been successfully prepared by Jing et al. via a facile solvothermal method under acidic conditions [231]. The metal organic framework acts as an effective photocatalyst for Cr(VI) reduction and can remove different aqueous contaminants with malachite green (MG) as a scavenger.

#### Stability of TiO_2_-modified photocatalysts

The stability of a photocatalyst is considered as an important aspect for its industrial application. A photocatalyst can be highly stable and can efficiently be industrially applied only when it is conveniently recovered from wastewater and reused effectively without any change in crystal structure, phase or weight. Hence, it is most essential to study the recoverability and recyclability of the photocatalyst. In this section, the stability of different TiO_2_-modified photocatalysts in terms of their regeneration ability and reusability in wastewater treatment for potential application in remediation of Cr(VI) has been addressed.

**Figure 12:**
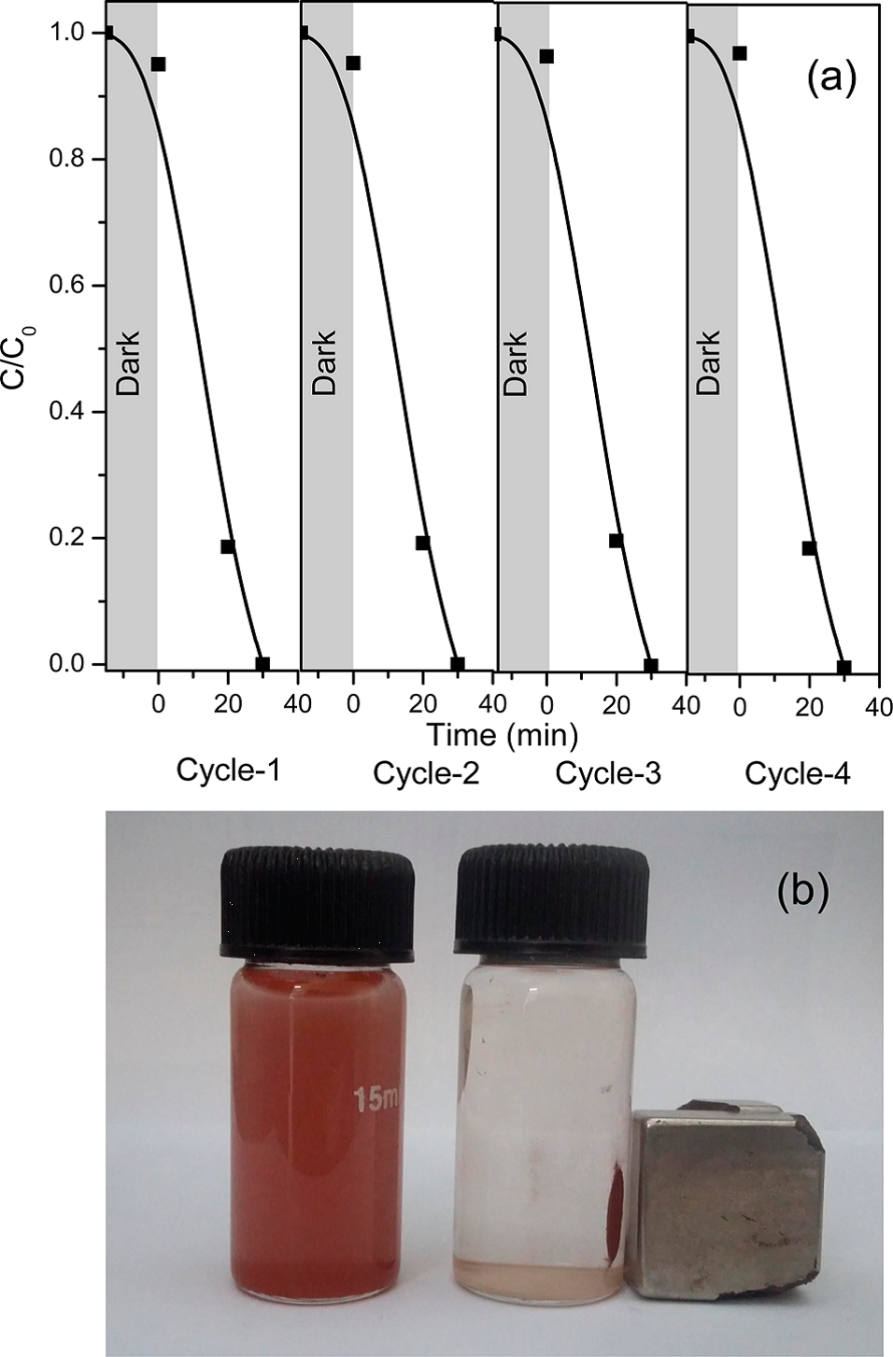
(a) Recyclability of TiO_2_/Fe_3_O_4_ towards photoreduction of Cr(VI) up to 4 cycles, and (b) images of the magnetic separation of TiO_2_/Fe_3_O_4_. Reprinted with permission from [158], copyright 2016 American Chemical Society.

Liu et al. have studied recycle tests up to five cycles for a RGO–TiO_2_ photocatalyst by taking fresh rhodamine B and Cr(VI) solution under stimulated solar light irradiation. Even after five cycles, there was no decrease of the performance of the catalyst, showing its high photostability [134]. RGO–Mn–TiO_2_ exhibited excellent stability with the high Cr(VI) removal efficiency of 96.61%, even after three cycles [137]. Wang and co-workers reported that graphene foam/TiO_2_ nanosheet hybrids could be promising in practical water treatment applications for removal of both Cr(VI) ions and organic dyes as these exhibited excellent recycle stability and easy recoverability [232]. The percentage removal of Cr(VI) was found to be 93%, 88%, and 80% for the first, second and third cycles, respectively, for TiO_2_/CdS films, indicating its high photostability [200]. Challagulla et al. reported that TiO_2_/Fe_3_O_4_ composite photocatalysts retained their efficiency towards reduction of Cr(VI) after the fourth cycle [158]. About 84% of it was recovered at the end of the fourth cycle. The recyclability of TiO_2_/Fe_3_O_4_ up to the fourth cycle towards photoreduction of Cr(VI) and its magnetic separation is shown in Figure 12.

The regeneration of the catalyst was confirmed by XRD for the original crystal structure, XPS for the oxidation state and binding energy of the core level elements, Raman spectroscopy for retention of phases, SEM-EDS for morphology and VSM analysis for saturation magnetization. Therefore, this photocatalyst can be considered as highly stable under reaction conditions for potential applications in wastewater treatment.

## Conclusion

The surface area, light absorption range, separation ability and transportation of photogenerated carriers are the parameters for controlling the performance of a photocatalyst in the remediation of Cr(VI). The modification of TiO_2_ results in the enhancement of the surface area, an increase in the light absorption range and the escalation of electron–hole pair separation, which in turn tremendously promote photoactivity towards reduction of Cr(VI). The high surface area of RGO causes fast adsorption of Cr(VI) onto RGO-modified TiO_2_ and facilitates transport of photoinduced electrons from TiO_2_ through the surface of RGO to suppress recombination of photogenerated charge carriers effectively. This results in an enhanced photocatalytic activity towards reduction of Cr(VI). The modification of TiO_2_ with RGO also extends the absorption range towards the red end of the visible spectrum. The concept of CDs in combination with TiO_2_ leads to better separation of photocarriers through the consumption of holes by in-situ-formed H_2_O_2_. Modifications with wide band gap semiconductor oxides like ZnO provide good contact with TiO_2_ and photocatalytic reduction of Cr(VI) was increased to a significant extent due to effective separation of charge carriers. However, the reaction is restricted to only the UV range. Narrow band gap semiconductors like metal oxides (e.g., Cu_2_O), mixed metal oxides (e.g., NiFe_2_O_4_) and metal sulfides (e.g., CdS, SnS_2_) form p–n heterojunctions upon coupling with TiO_2_ that created an inner electric field at the interface. The inner electric field formed provides a potential barrier which suppresses the recombination of charge carriers and facilitates the transport of photoelectrons for reduction of Cr(VI). As a result, the degree of photoreduction of Cr(VI) was remarkably increased. Furthermore, the heterojunction lowered the band gap energy between Ti 3d and O 2p states of TiO_2_ due to which light absorption of the coupled photocatalyst was extended to the visible region of the solar spectrum.

Dyes are used for sensitization of solar light to the surfaces of TiO_2_-based semiconductors to enrich light harvesting. In this review, porphyrin, xanthane and azo dye based sensitization with TiO_2_ catalysts are briefly discussed. The most effective method which was recently implemented is surface plasmon resonance metal induction in TiO_2_ through hot electron transition. Au, Ag and Pt metals having plasmonic properties coupled on TiO_2_-based plasmonic photocatalysts are discussed. Enhanced photoactivity has been reported when bimetallic (plasmonic and other metals) catalysts are utilized.

**Figure 13:**
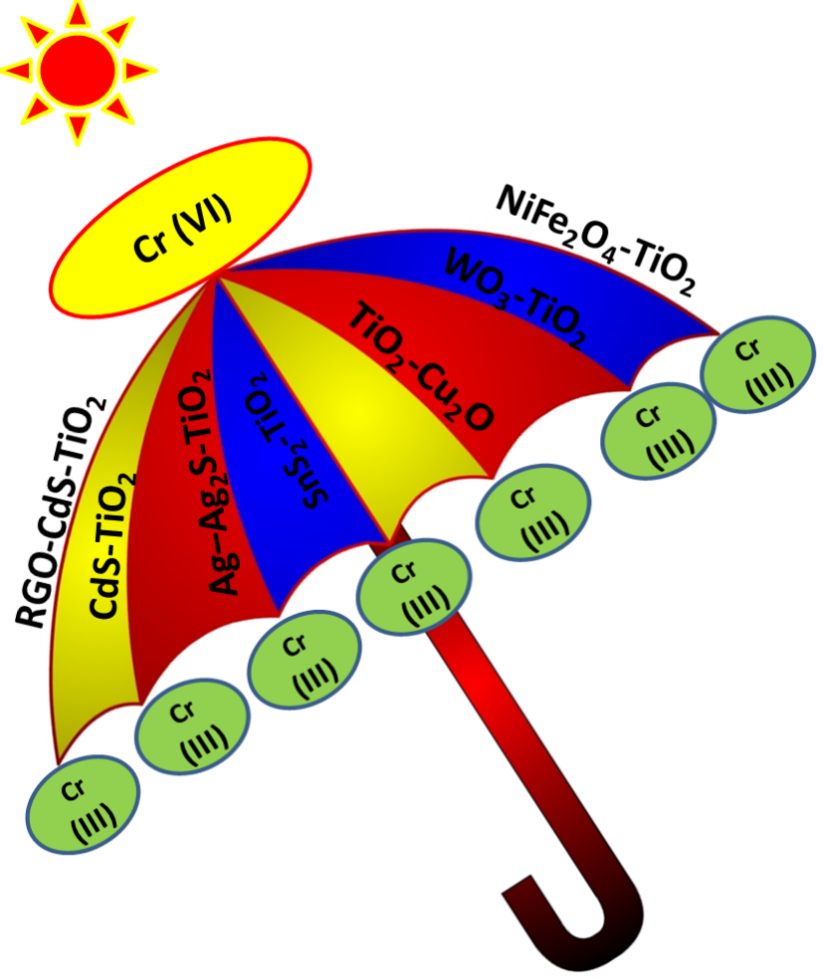
Summary of narrow band gap semiconductors that can be combined with TiO_2_ for effective photocatalytic reduction of Cr(VI) through solar energy conversion.

The photocatalytic reduction of Cr(VI) also depends on controlling experimental parameters like the pH of the solution, concentration of Cr(VI), catalyst dose and irradiation time of the photocatalyst. It was evident from Table 1 that the optimum conditions for maximum reduction of Cr(VI) varied from catalyst to catalyst, and hence, photocatalytic activity cannot be compared. However, a range of optimum conditions for maximum reduction can be listed for further research in this field. These optimal conditions are as follows: pH ≤ 5.5, initial Cr(VI) concentration 5.0–50.0 mg L^−1^, catalyst dose 0.2–1.0 g L^−1^ and irradiation time 15–360 min. A complete reduction of Cr(VI) was carried out over WO_3_/TiO_2_ NTs,TiO_2_/Cu_2_O, CdS@TiO_2_, RGO–(CdS nanowire)–TiO_2_, SnS_2_/TiO_2_ and Ag–Ag_2_S/TiO_2_ composite photocatalysts by harvesting visible light. In addition, the stability of the photocatalyst is an important factor as far as industrialization of the process is concerned. A few studies revealed the adequate stability of a modified TiO_2_ photocatalyst for efficient regeneration and reusability. NiFe_2_O_4_-modified TiO_2_ can also be considered as a promising photocatalyst not only due to its high photocatalytic activity towards reduction of Cr(VI) under visible light illumination, but also due to its good magnetic behavior that facilitates its separation from treated solution by the application of an external magnetic field. Figure 13 represents the combination of narrow band gap semiconductors with TiO_2_ for effective photocatalytic reduction of Cr(VI) under solar energy conversion.

The combination of excess RGO, metal oxides, mixed metal oxides and metal sulfides with TiO_2_ resulted in the development of new recombination centers, which facilitates recombination of electron–hole pairs leading to poor photocatalytic activity.

Although photocatalytic reduction of Cr(VI) over modified TiO_2_ aims to be an environmentally benign and energy sustainable process, it faces some challenges regarding its practical applicability. These are described as follows.

1. Despite the extension of the light absorption range of TiO_2_ from the UV to the visible region by its modification with carbon-based smart materials, metal sulfides, noble metals or the formation of p–n junctions using narrow bandgap metal oxides, the utilization of the complete solar spectrum is yet to be achieved for harnessing solar light for the photoreduction of Cr(VI).
2. The use of noble metals such as Ag, Au, Pt are costly, and hence, their use in designing the photocatalyst will be expensive. It is a challenge to modify TiO_2_ with relatively low cost metals with the retention of the light absorption ability and properties of separation of charge carriers.
3. The remediation of Cr(VI) through photocatalysis is restricted because of loss of weight during recycling of the photocatalyst. Although a few studies reported stability of modified TiO_2_ up to five cycles, the number of reuse cycles must be increased without loss in weight in order for widespread commercialization of the process.
4. During photocatalytic reduction, Cr(VI) species are converted to Cr(III) ions, which are deposited on the surface of the photocatalyst; the resulting Cr(OH)_3_ shields the active surface sites and hinders the rate of reduction of Cr(VI). As a result, the efficiency of the photocatalyst is largely deteriorated during the recycling process. Therefore, the major challenge in photocatalytic reduction of Cr(VI) is to remove/inhibit the formation of Cr(OH)_3_ on the photocatalyst surface.
5. Attention must be given to investigate remediation of Cr(VI) from wastewater, whereby the pH normally lies above that of the synthetic solution that is commonly used for photocatalytic reduction.

The future prospective of this review depends on the selection of appropriately modified TiO_2_-based photocatalysts for enhanced photoactivity in the complete solar spectrum. The modification of TiO_2_ with surface plasmon materials induces hot electron generation and injection to the CB of TiO_2_ semiconductors for better charge separation as well as light harvesting, leading to higher photocatalytic efficiency. The focus on cost effectiveness should be emphasized for use of plasmonic photocatalysts such as Al, Bi, and Cu instead of Pt, Au etc. Another alternative is to couple a RGO hydrogel and NiFe_2_O_4_ with TiO_2_ to set up a photocatalytic system with a low charge recombination rate and fast photoreduction of Cr(VI) by harvesting solar energy. This system will have the major advantage of easy magnetic separation of the catalyst from the treated solution. Efforts must also be given to increase the stability of the photocatalyst for long run without decrease in efficiency. Overall, a comprehensive attempt by the research community in the relevant fields should be made to overcome the differences in results between lab-scale research and large-scale industrial applications. Hopefully, the present review will provide a stepping stone to accelerate research in developing highly efficient photocatalysts with significant stability for remediation of Cr(VI) from wastewater through photocatalysis.
